# Supporting long-term condition management: a workflow framework for the co-development and operationalization of machine learning models using electronic health record data insights

**DOI:** 10.3389/frai.2024.1458508

**Published:** 2024-11-12

**Authors:** Shane Burns, Andrew Cushing, Anna Taylor, David J. Lowe, Christopher Carlin

**Affiliations:** ^1^Lenus Health Ltd., Edinburgh, United Kingdom; ^2^Departments of Respiratory and Emergency Medicine, Queen Elizabeth University Hospital, NHS Greater Glasgow and Clyde, Glasgow, United Kingdom

**Keywords:** machine learning in healthcare, long-term condition management, risk prediction, model operationalization, electronic health record data, workflow framework

## Abstract

The prevalence of long-term conditions such as cardiovascular disease, chronic obstructive pulmonary disease (COPD), asthma, and diabetes mellitus is rising. These conditions are leading sources of premature mortality, hospital admission, and healthcare expenditure. Machine learning approaches to improve the management of these conditions have been widely explored, with data-driven insights demonstrating the potential to support earlier diagnosis, triage, and treatment selection. The translation of this research into tools used in live clinical practice has however been limited, with many projects lacking clinical involvement and planning beyond the initial model development stage. To support the move toward a more coordinated and collaborative working process from concept to investigative use in a live clinical environment, we present a multistage workflow framework for the co-development and operationalization of machine learning models which use routine clinical data derived from electronic health records. The approach outlined in this framework has been informed by our multidisciplinary team’s experience of co-developing and operationalizing risk prediction models for COPD within NHS Greater Glasgow & Clyde. In this paper, we provide a detailed overview of this framework, alongside a description of the development and operationalization of two of these risk-prediction models as case studies of this approach.

## 1 Introduction

The NHS and other healthcare systems face various long-term challenges, many of which have been exacerbated by the impacts of the COVID-19 pandemic on existing financial strains and waiting list backlogs (Cutler, 2022; Khan, 2016). A key issue is the increasing number of individuals affected by long-term conditions (Atella et al., 2019; Holman, 2020). Long-term conditions (often referred to as chronic conditions) are defined as conditions which cannot currently be cured and require continuous medical treatment, such as cardiovascular disease, chronic obstructive pulmonary disease (COPD), asthma, and diabetes mellitus (NHS, 2023). These conditions present a major mortality and morbidity burden, with cardiovascular disease and COPD alone accounting for 17.9 million and 3.32 million global deaths per year, respectively, (World Health Organization, 2023). Within the UK, long-term conditions account for 70% of inpatient bed days and are the leading cause of premature mortality (Department of Health, 2012; NHS, 2019). Transformation of the models of care for these conditions to ensure proactive personalized preventative management is essential to improve the quality of life of patients, prevent avoidable and costly hospital admissions, and reduce premature mortality (NHS, 2019).

The use of machine learning (ML) has the potential to support care model transformation and alleviate pressures on healthcare systems. The proposed benefits of ML for use in the long-term condition context include time and resource savings from the automation of tasks outside of patient facing care, and the aggregation and analysis of cohort and population-level data to support early accurate diagnosis, triage, and treatment selection (Davenport and Kalakota, 2019; Javaid et al., 2022). There have been many prospective clinical use cases for ML investigated using various data sources including structured data such as medical history data stored in electronic health records (EHRs), semi-structured data such as time series data derived from wearable medical devices and unstructured data such as medical imaging data (Habehh and Gohel, 2021). The existing digital pathway for image acquisition, reconstruction, interpretation, and reporting has meant that a range of diagnostic ML models using imaging have been deployed in a live clinical environment as part of active screening, workflow assistance and decision support programs (Koh et al., 2022). However, despite extensive reports of clinical ML models in the academic literature, there has otherwise been limited translation of these models into tools used in a live clinical context. Lack of clinical user co-design and oversight, and absence of planning for model operationalization are typical gaps in healthcare ML model development which have hampered the use of ML models in clinical care (Bastian et al., 2022).

Given the need to evolve working practice to a more collaborative, adoption-focused approach, we have defined a workflow framework for the co-development of ML models trained using routinely collected data derived from electronic health records (EHRs). This framework is based on the first-hand experience and learnings our team has acquired from developing and operationalizing models to support the management of patients with COPD. The framework describes a collaborative working process between clinical and technical teams, spanning from establishing a multidisciplinary team to the investigative use of models in a live clinical environment. This work aims to provide a blueprint for multidisciplinary teams to navigate the complex landscape of model development and operationalization, with the aim of increasing the number of ML tools available to clinical teams and facilitating the generation of real-world evidence on the feasibility, safety, utility, and acceptability of using ML models to improve long-term condition management. Strategies to address wider issues that have confounded the application of ML approaches within a live clinical setting, such as the lack of interpretability of many ML models (Gill et al., 2023; Pierce et al., 2022) and the risk of models underperforming in subpopulations within the intended inferencing population (Fletcher et al., 2020) will be explored. Additionally, this paper presents two case studies documenting the development and operationalization of a 12-month mortality risk-prediction model and a 90-day readmission risk-prediction model by our multidisciplinary team. Details of the considerations made at each stage and the lessons learned throughout the process are included to demonstrate how the principles described in the framework can be applied in practice.

## 2 Workflow framework

**Figure 1:**
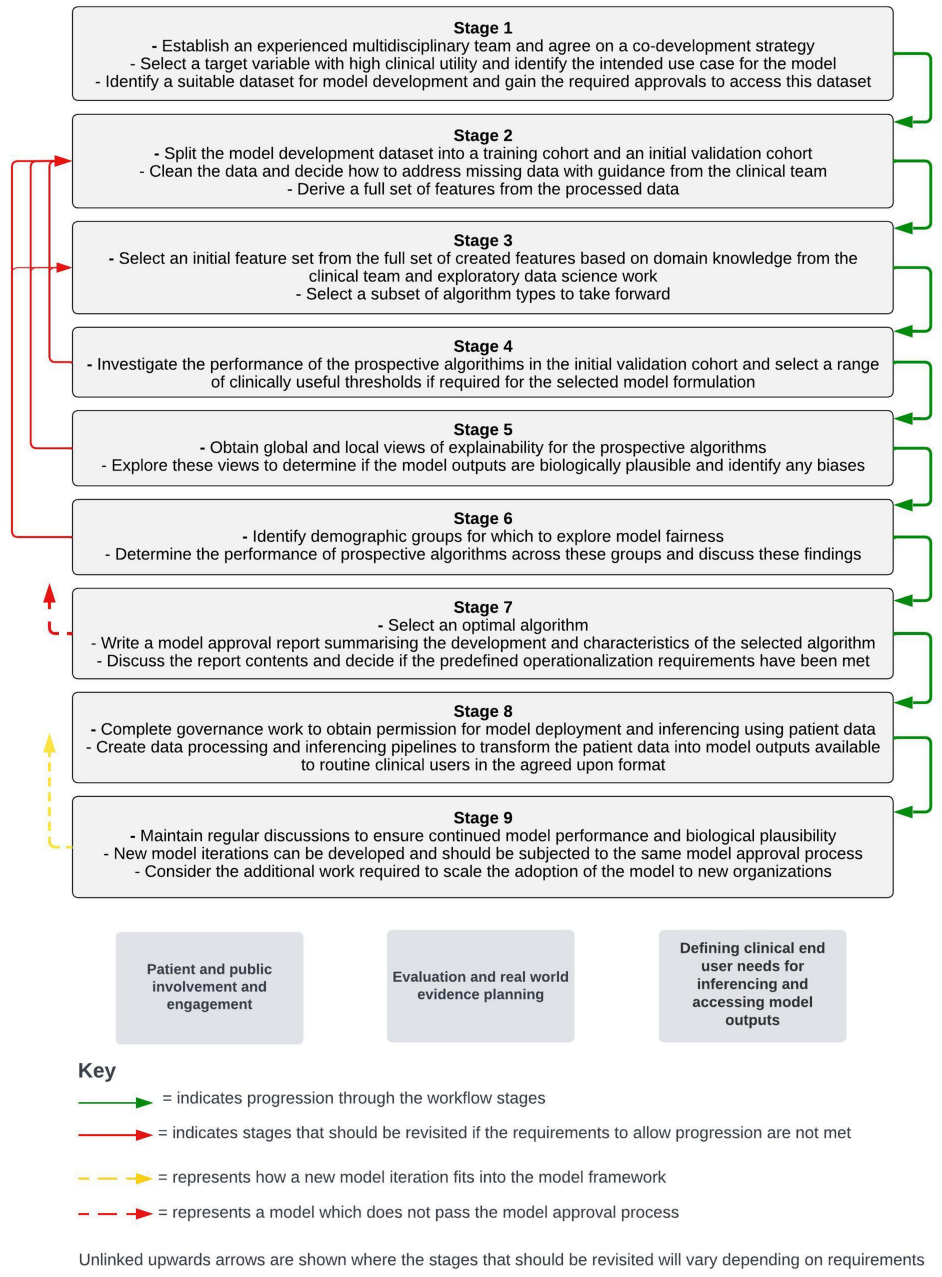
Stages of a workflow framework for co-developing and operationalizing machine learning models from structured electronic health record data. Workstreams which sit outside of the stepwise framework but are key to the adoption of machine learning models in live clinical care are shown below the stepwise stages.

This framework is informed by the direct experience of our multidisciplinary team in the operationalization of risk-prediction models for COPD as part of the “DYNAMIC-AI” clinical investigation (NCT05914220). The framework has been broken down into nine distinct stages to highlight the considerations and requirements at each point of this process. To maintain simplicity, this framework will assume that a supervised machine learning approach is used and that only the structured data contained within EHRs is used to create features. A visual summary of each stage of the workflow framework is shown in Figure 1.

Outside of the immediate model development and operationalization process, parallel workstreams will be required for the adoption of models into a live clinical environment. These workstreams include patient and public engagement and involvement activity, defining routine clinical user needs for inferencing and accessing model outputs, and planning around evaluation and real-world evidence generation following model operationalization. The timing of different components of these workstreams relative to the stepwise model development and operationalization process will vary with different approaches. As a result, a separate section detailing considerations around these workstreams is included after the stepwise stages presented below.

### 2.1 Stage 1: establishing a multidisciplinary team, identification of a target variable, and model development dataset identification

The first step of model development should be identifying a multidisciplinary team (MDT). This MDT should consist of a clinical and a technical team. The clinical team should include clinicians who are experts on the target long-term condition or conditions of interest, as well as expert and routine clinical users who should be involved in the implementation of the developed model(s) in a real-world clinical environment. The technical team should include members with data science, data-engineering, cloud-engineering, and potentially software development experience if a novel user interface is going to be required to share the model outputs. The clinical team will provide detailed knowledge of the disease indication, provide context on current treatment paradigms and outline the problem that developed model(s) and associated insights can potentially address, define where model(s) and insights would fit within current clinical workflows, provide feedback on the biological plausibility of model outputs, and assist with the transformation of workflows and clinical pathways. It is critical that there is clinical oversight throughout the process to ensure that any decisions made will lead to a model with maximal utility, that could feasibly be integrated into clinical care. The technical team will train and validate models, set up the required data pipelines for data processing and model inferencing and create a mechanism for clinical users to routinely review model outputs. At this point a co-development strategy should be outlined so it is clear what the stages of the project are and what the forums are for MDT discussions throughout the project. To ensure the safe and efficient running of the project and to minimize delays, the co-development strategy should also outline the processes required to adhere to information governance standards and specify the points in the process where input and approval will be required from information governance responsible officers.

Once a co-development strategy has been established, the intended target variable should be identified. For example, this may be the optimal therapy for an individual, the likelihood that an individual would benefit from a particular therapy, or the risk of an individual experiencing an adverse event such as hospitalization. To ensure maximal clinical utility, the clinical team should make this decision, incorporating the views of patients with the target condition(s) of interest on their care priorities. For risk-prediction model formulations, consideration should also be made to the timeframes for prediction, as the time window from the point of inference and identification of high-risk patients, to the point where clinical action could be taken must be aligned to appropriate timelines for intervention. The point in current workflows where risk-prediction models are intended to be implemented and the intended use case for the models should be outlined, with an understanding that this may evolve within the process as insights from model development and refinement will potentially be combined with evolving transformation of care processes and clinical workflows. The differences in care models and workflows between healthcare systems, and how that may impact the utility and adoptability of developed models by other organizations should be considered at this stage.

Following this, a suitable model development dataset will need to be identified by the clinical team, and the data scientists in the technical team will need to be provided with safe access to this data. The dataset should contain as many potentially informative fields as possible related to the medical histories of the individuals within the dataset. The provided data will need to be in a de-identified format, but robust data linkage strategies implemented by data controllers can allow for information from different data sources to be collated for individual patients. The approach to data linkage established at this point should also consider model inferencing in a live clinical environment at a future stage. The cohort within the dataset should be sufficiently large for the purposes of training ML models and should be representative of the population the model is intended to run inferencing on in terms of key demographic factors and disease severity. Statistical tests and data drift tools can be applied when there is uncertainty around this. It is necessary to discuss any data redactions and censoring that has been applied to the model development dataset with the data controllers as certain data may have been removed such as data related to extremely rare or sensitive diagnoses or prescriptions. Any redactions and censoring can then be discussed between the clinical and technical teams to plan any required adaptations to feature engineering and selection. Providing the data scientists with access to the dataset will likely require significant governance work including completing detailed applications to bodies such as local privacy advisory committees within the NHS, justifying the need for data access and defining the scope of the planned activities. Additionally, virtual environments for the data science team to train the models within will likely need to be created, depending on data controller requirements. This may result in delays which should be accounted for in any project time scales.

Once the data scientists in the technical team have access to the training data, exploratory work should be conducted to determine: the available data fields for model training, the distribution of the target variable, the breakdown of the cohort overall in terms of disease severity and demographic factors, the extent of missing data across the dataset and the relatability of the dataset to other organizations. The insights gained from this exploratory analysis should be shared with the clinical team and discussions based on this exploratory work should take place. The clinical team should also share insights around clinical workload, capacity levels and resource levels with the technical team at this point as this will provide insight into the anticipated use of the model(s) within a live clinical environment. These discussions will inform the next steps taken in model development and the desired characteristics of any developed models.

### 2.2 Stage 2: train-test splitting, data preprocessing, and feature engineering

The individuals included in the model development dataset should be split into a training cohort and a cohort to be used for initial evaluation of the model on unseen data. If multiple index dates per individual patient are used in model development, it should be ensured that train-test splitting is conducted at a per-individual rather than at a per-row level. This avoids the potential for individuals to appear in both the training and validation datasets. It should also be ensured that the training and validation datasets have a similar distribution of the target variable (particularly if there is significant class imbalance) and that the validation dataset is broadly representative of the overall dataset with respect to key demographic factors. The same pre-processing and feature engineering steps described below should be conducted on both the training and the initial validation datasets.

One significant drawback to EHR data as a data source for training ML models is the frequency of data quality issues such as missing timestamps, values outside of biologically plausible ranges and duplicated data (Sauer et al., 2022). However, many of the data quality issues can be addressed by pre-processing of the data. Therefore, it is vital that this process is as rigorous as possible, as the quality of the input data will directly impact the reliability of the model outputs. As part of this process, the clinical team should discuss the minimal and maximal biologically plausible values for different fields with the data scientists in the technical team so that values contained in the fields significantly outside of these given ranges can be identified as mis-entered data and removed. Additionally, duplicate recordings should be removed, adhering to specified criteria from the clinical team in what would constitute a duplicate for different fields within the dataset. It should also be ensured that all measurements for each field have been recorded in consistent units prior to deriving features.

Another consideration with the application of ML to EHR data is the high levels of missingness in fields of interest for some individuals in the dataset. This is a result of different individuals having different historical healthcare contacts and different information being collected during different healthcare contact types. For example, certain blood tests would only be taken in specific contexts and therefore not all individuals in the dataset would have recorded data for the field related to that test. Multiple considerations must be considered when determining how to address missing data. Firstly, some ML algorithm implementations are unable to handle missing data entirely and so using an imputation method to derive values for the missing fields or removing fields with excessive missingness may be required depending on the selection of algorithms that are being considered at this stage. Another consideration to make is the context for which the data is missing, which may not be clear to the data scientists processing the data. To use the blood test example again, it may be the case that differences in testing frequency exist across different geographies due to local testing protocols, which could pose challenges if the dataset encompasses data from a wide geographic area. If missing data is not imputed it is therefore possible that the model learns the context of data being missing (i.e., treatment in a particular geography) and starts to associate this with the target variable rather than the value of the measurement (Sauer et al., 2022). As a result, discussions between the clinical team and the data scientists in the technical team on the context for which certain data fields would be taken and the optimal way to deal with missing data for different fields are required. The possible biases introduced with different approaches to handling missing data, and a cutoff for missingness before fields are removed should be considered. Other general data pre-processing for structured data such as normalization and categorical encoding may be required depending on the fields available within the EHR dataset and the algorithm types under consideration.

Once the initial preprocessing has been completed features can be derived from the training dataset. Feature types may include aggregate counts of events, procedures, or therapies over a set time window, and features specific to the most recent occurrence of an event, test, or procedure. Target encoding—a process which takes the average value of the target variable for each category- can be used for binary classification formulations where there is a vast range of possible categorical values for a particular field which is common in EHR data. If target encoding is conducted, the average values for each category should be determined in the training dataset and the resulting encodings applied to both the training and validation datasets to avoid data leakage. The clinical team should help inform the scope of the features that are created by sharing domain knowledge on typical patient trajectories and treatment paradigms for the target condition.

### 2.3 Stage 3: initial feature selection and exploration of algorithm types

After a full feature set has been derived from the dataset the clinical team should highlight markers associated with the target variable using domain knowledge on the target condition. In the case of a risk-prediction model the clinical team may also want to highlight specifically actionable features from the full feature set, to ensure that these features are included, so that modifiable risk factors are presented at the point of inference. The initial feature set can then be determined by combining these features, with features associated with the target variable identified by the data scientists in the technical team through exploratory analysis. At this stage, the performance of different algorithm types should be explored to inform which to take forward. The data scientists in the technical team may wish to apply upsampling and downsampling techniques prior to model training where notable class imbalance is observed. Outside of the initial model performance in the training dataset, several attributes of prospective algorithms such as sensitivity to noise and outliers, ability to tune model hyperparameters, interpretability, and ability to handle missing data should be considered when deciding which algorithms to take forward. This decision necessitates input from various members of the technical team, as different algorithms may have different resource requirements for deployment.

### 2.4 Stage 4: initial evaluation of model performance on an initial validation dataset

At this stage, the data scientists in the technical team should evaluate the performance of the prospective algorithms on an unseen initial validation dataset to identify if the models have been overfit to the training dataset. If the model has significantly poorer performance in the validation set than the training dataset, overfitting has occurred. In this instance the initial steps that can be taken are reducing the complexity of the prospective algorithms and reducing the number of features in the model. If these steps are unsuccessful, alternative features can be selected from the full feature set or derived from the training dataset.

A range of means of evaluating model performance can be employed. Common evaluations for classification problems include assessing the area under receiver operating characteristic and precision-recall curves, confusion matrices, and accuracy and F1 score metrics. Common evaluations for regression problems include variants of the mean squared error and R-Squared metrics. In the case of classification problems, the evaluation metrics focused on should consider the class balance of the problem. This is particularly relevant for model formulations to predict rare adverse events such as mortality. For context, if the negative class was the true class in 95% of instances, a model could achieve an accuracy of 95% by predicting the negative class in every instance. In this case, the accuracy metric makes the model appear very performant, but the model would offer no insight or clinical value. Therefore, metrics that assess the ability of the models to identify the minority class should be focused on where class imbalance is presented. The performance metrics of the prospective algorithms and the context of how these metrics can be interpreted should be shared with the clinical team. The impact of altering the decision threshold on performance metrics and the manageability of actioning the model decision at different thresholds should also be discussed at this point to identify a range of clinically useful thresholds.

### 2.5 Stage 5: obtaining global and local level explainability

After performant models have been identified, it is critical for both the data scientists and the clinical team to gain a better insight into the inner workings of the candidate models. Specifically, how the inputs to the model are used to generate the output at both a global (cohort level) and individual level (patient level) must be understood to bolster clinical, and public support of the use of ML models in a clinical environment (Musbahi et al., 2021; Young et al., 2021). Additionally, including individual explainability views for risk-prediction models can allow for potential intervenable risk factors to be identified at the point of model inferencing, significantly increasing the utility of the model outputs and the efficiency at which management can be optimized. Sessions should take place between the clinical team and the data scientists in the technical team, where global and illustrative local level explainability views for the candidate models are shown. In these sessions it should be determined if the model outputs are biologically plausible and if there are any undiscovered biases affecting the prospective models. These biases can be identified by discussing if any of the features are interacting with the target variable in an unexpected way. If biases are identified within any of the features, the clinical team should consider the data collection process for the biased feature to understand what the source of the observed bias may be. Through these sessions it can be determined if the feature can be reformulated, or if the feature should be removed altogether. If biases are observed that cannot be rectified by altering or removing a small number of features, this may reflect a bias in the model formulation or in the model development cohort inclusion criteria requiring more substantial changes.

For some algorithm types, the influence of each of the features on predictions can be easily interpreted. For example, with logistic regression this can be ascertained by the model coefficients. However, the internal workings of other algorithms such as neural networks are less transparent due to their complexity, making it very difficult to interpret the relationship between the input features and the model output (Fung et al., 2021). Unfortunately, it is often the case that more complex, and less inherently explainable algorithms are more performant than simpler more interpretable algorithm options. If there is a significant difference in performance between the prospective algorithms with differing levels of inherent explainability, model agnostic frameworks such as SHAP present an alternative means to obtain a measure of the impact of each of the model features on the model output for individual predictions and at a global level (Lundberg and Lee, 2017). Regardless of the means used to obtain explainability, the data scientists in the technical team should ensure that time is allocated to talk through how any explainability metrics can be interpreted as it is likely that these will be unfamiliar to clinical teams.

### 2.6 Stage 6: assessing model performance across different demographic groups

A major concern with the introduction of ML models into a live clinical setting is the potential to exacerbate existing healthcare inequalities related to factors such as socioeconomic status (SES), sex, ethnicity, and age. Poorer model performance amongst specific subpopulations is often a result of the model development dataset not being representative of the population for which the model is intended to run inference on (Sunarti et al., 2021). Although EHR datasets capture data for all individuals interacting with healthcare systems, variable performance of some ML models trained on EHR data by socioeconomic status (SES) and ethnicity have been reported (Gianfrancesco et al., 2018; Juhn et al., 2022). It has been suggested that this is a result of reduced representation of people with lower SES and people from minority ethnic groups within EHR data due to factors such as individuals in these groups being less likely to attend a consistent place of care and being more likely to rely on safety net care such as emergency department care (Cardet et al., 2018; Flores et al., 2009; Juhn et al., 2022). Separately, upon investigation, some published ML models have poorer performance in females compared to males. For example, a study investigating the relative performance of various diagnostic classifiers for liver disease trained on an extensively used model development dataset, found that false negative rates were higher in females across all classifiers and that some of the laboratory test features key to model decision making had a reduced predictive power in females (Straw and Wu, 2022). It is therefore critical to look at the performance of candidate models across a variety of demographic groups to investigate if the model performance is significantly different between demographic groups. The exact demographic groups that are looked at will depend on the data available and the demographic breakdown of the population that the model is intended to run inference on. Data fields showing information related to these demographic groups should be included amongst the model features to avoid omitted variable biases and so that the impact of these features can be explored through explainability views.

A session should be scheduled for the clinical team and the data scientists in the technical team to discuss the performance of the prospective models across the different demographic groups of interest. These may include factors such as age, sex, SES, and ethnicity. Through discussions with the clinical team, it can be identified if bias exists (i.e., if the model has improved performance in one subgroup compared to another when looking at a particular demographic factor) and if the bias would be considered unfair or unethical. To be able to properly compare fairness between different algorithm types, we recommend evaluating a selection of relevant performance metrics across the different demographic groups of interest. It should be noted that bias within an algorithm is a mathematical reflection of a difference in performance between two subgroups in any direction and is independent of ethics. Determining if this bias is ethical or fair requires assessing the impact of the bias against a set of ethical or legal principles (Fletcher et al., 2020). It may be determined that although bias exists, it does not contravene these principles and so does not affect the clinical acceptability of a prospective model.

It is also worth noting that the threshold for decision making will impact the performance metrics and therefore the bias and fairness of prospective models. Performance metrics could be similar across demographic groups at one threshold but vastly different at another. Therefore, model fairness should be considered when selecting a decision threshold from the range of clinically useful thresholds. If there are unacceptable differences in model performance between different demographic groups at all clinically useful thresholds, the earlier stages of the framework should be revisited, and new features and algorithm types should be explored. Another option is to retrain the model using a fairness-aware loss function, although this can lead to a drop in performance across all demographic groups and therefore may not be a suitable option in all cases.

### 2.7 Stage 7: model selection and initial model approval

At this stage, the remaining candidate algorithms should be compared to identify and select an optimal model to bring forward. This decision should consider model performance overall and across demographic subgroups in the initial validation set following light hyperparameter tuning and a further round of feature selection to increase model performance across the different candidate algorithms. The selected model should then undergo more rigorous hyperparameter tuning to maximize model performance. Subsequently, the optimized model should be subjected to a formal documented model approval process to determine the clinical acceptability of the model, requiring the preparation of a model approval report by the data scientists in the technical team. The report should contain details of the problem formulation and the training and validation process, a description of the model development dataset (describing the cohort included, date ranges covered and the fields in the dataset used to derive the input features), a summary of the feature engineering process, a list of the features included in the selected model, high-level information on the algorithm type used and key term definitions, details of the performance metrics of the selected model both overall and across different demographic groups, and global and local explainability views for the selected model. The model approval report should be shared with the clinical team, with a structured review session arranged between the clinical team and the data scientists in the technical team to determine if the requirements for the model to be adopted into a live clinical environment have been met. If the model passes the approval process, operationalization work can begin, otherwise the earlier stages in the framework should be revisited to create a new model variant with the required changes indicated by the clinical team. This new variant would then undergo the same documented model approval process.

### 2.8 Stage 8: model operationalization

Operationalizing a model into a live clinical environment will require significant governance work and sufficient time should be allocated within the project time scale for this. The exact steps will vary according to local processes in the target deployment site. Data processing agreements between the institution providing data and the institution accessing the data will likely be required and there may be additional checks and assessments conducted given the sensitivity of healthcare data. It should be ensured that any redactions applied to the training dataset are also applied to the live data, to match the model training context. The methods by which data is exchanged will likely necessitate the deployment of or updates to IT infrastructure, requiring the approval of the relevant change control board. When the relevant governance work has been completed, the latest data for the patient population will then need to be transformed from the format it is stored in into the final feature set decided on during model development. To achieve this, data processing pipelines will need to be created by the technical team to conduct the preprocessing and feature engineering steps in a secure environment. Following this, the technical team will need to establish a pathway for running inferencing on the processed data and outputting model scores and explainability views in the agreed upon format. If deployment is being undertaken as part of a clinical trial, which is very likely in the current regulatory landscape, an approved clinical investigation plan, associated ethics approval and a notice of no objection from the relevant regulatory body or equivalent are pre-requisites.

### 2.9 Stage 9: post-operationalization monitoring and scale-up considerations

After the point where the first model iteration has been operationalized, regular discussions should take place between the clinical users and the technical team to ensure that the model continues to be performant and that the model explainability views continue to be biologically plausible. Model performance may change over time due to factors such as changing EHR recording practices, new interventions being developed, and changes to disease management approaches (triggered by or independent of model insights), necessitating the development of new model iterations. The data scientists in the technical team can develop subsequent model iterations to respond to this and to enact feedback from the clinical users. These updated model iterations would then be subjected to the same model approval process that was described in stage 7 (with explicit reference made in the model approval report to any changes made since the previous model iteration) and could be operationalized by altering the data processing pipelines created in Stage 8. In a clinical trial context, the protocol should prespecify whether models will be used unchanged, adapted on a schedule, or adapted flexibly within the investigation period. The implications of each approach on trial analyses should be considered during protocol development, requiring trial methodologist and statistician input.

In addition to this supervision and improvement activity, this is the stage at which external reporting of implementation and effectiveness evaluations should be planned and knowledge exchange with other organizations who wish to adopt the model as part of the scale-up of use should be incorporated. Scale-up of the model will either require demonstration of transportability through external validation in another dataset reflective of the intended target population for scale-up, or demonstration of performance in the target population itself (la Roi-Teeuw et al., 2024). The model may need to be updated based on the requirements of the clinical teams working in the scale-up sites, the available data for inferencing in these new sites, and the governance and regulation in the scale-up sites. The ease of scaling the model to a new site will depend on the similarity of clinical workflows, available data, and population characteristics between the new site and the original operationalization site. Input from scale-up sites as well as outputs from the implementation and effectiveness evaluations would be expected to inform model improvements and refinements.

### 2.10 Parallel workstreams: defining routine clinical user needs for inferencing and accessing model outputs, patient and public involvement and engagement activity, and evaluation planning

Sessions should take place to decide on the details of the model inferencing process and how the outputs of the model can be viewed by the clinical team responsible for actioning the model. The criteria for an individual to be included in model inferencing will need to be determined and the frequency at which new inferencing runs are required will need to be considered. Critically an appropriate way of sharing model scores and explainability insights with the members of the clinical team responsible for actioning the models will need to be agreed, factoring in the technical and computational resource requirements for different options. In some cases, development work will be required to create a novel co-designed user interface, or alternatively a much simpler approach could be taken depending on the organizational needs of different clinical teams.

Patient and public involvement and engagement is vital to ensure alignment between patients and clinical teams on care priorities and what the potential role of models in clinical pathways should be and the acceptability of the model development and operationalization process. Input from patients and the public requires consideration across model development, operationalization and improvement stages particularly around how models are used to inform clinical care, fairness considerations, and exploring any unintended consequences of adopting ML models in clinical care. Reflective structured patient and public engagement across the model development processes should be planned and resourced. This will often include direct user research (semi-structured individual or group interviews, surveys), review of and reference to published materials, focused co-design sessions with patients, and incorporation of patient-focused endpoints within the model operationalisation evaluation plan.

For an ML application to be used in clinical care in the long-term and scaled, real-world evidence of the feasibility, acceptability, safety, utility, value and sustainability of using the model as part of clinical care must be obtained. An evaluation plan should be established in parallel with model development and operationalization. The feasibility of generating model insights from patient data in live inference runs, the acceptability of using patient’s data to inform their care, and the safety of this approach should first be determined. Further analyses exploring the utility, clinical effectiveness and potential cost effectiveness of ML model use on care pathway transformation will then be required. This may partly be acquired at the initial operationalization stage, and then augmented as further real-world experience grows at the post-operationalization scale up stage. It is essential to ensure that all required data for evaluation and supervision such as clinical user experience, clinical actions taken based on model insights, and prospective model performance will be captured and accessible for analysis. Advice from a health technology assessment body and/or associated academic partners is advised to ensure appropriate implementation and evaluation planning at this point. In the UK, the Health Research Authority (HRA) and the Medicines and Healthcare products Regulatory Agency (MHRA) have recently published guidance on these considerations (Health Research Authority, 2024; Medicines and Healthcare products Regulatory Agency, 2024).

## 3 Case studies

### 3.1 Background

COPD is a common, treatable respiratory disease with acute exacerbations being responsible for a substantial proportion of disease burden, adverse outcomes, and healthcare expenditure. In the UK, COPD is responsible for 30,000 deaths per year (Snell et al., 2016) and is the second largest cause of emergency admissions (National Institute for Health and Care Excellence, 2019). Assistive technologies such as predictive artificial intelligence (AI)-driven insights could provide individualized, accurate and, where possible, actionable areas for clinical intervention in COPD, reducing disease and healthcare burden, and driving a shift from reactive to proactive medical care. Given this context, using funding support from an Accelerated Access Collaborative/NIHR Artificial Intelligence in Health and Care award, our multidisciplinary team consisting of clinicians from the respiratory innovation team in the NHS Greater Glasgow and Clyde (NHS GG&C) health board in Scotland and a technical team from Lenus Health worked collaboratively to develop and operationalize mortality, readmission and exacerbation risk stratification models to support clinical decision making. In this section we present the development and operationalization of our 12-month mortality prediction model and our 90-day respiratory related readmission prediction model as case studies.

For both models, scores are reviewed following an inference run by members of the COPD multidisciplinary care team in NHS GG&C. Model scores at or above the predetermined thresholds prompt review of an individual’s healthcare records alongside explainability insights. COPD management optimization considerations are then discussed for highlighted participants and actioned if appropriate. Extensive patient and public engagement work was undertaken as part of the model development and preparation for the clinical trial. We noted published patient priorities for COPD research (Alqahtani et al., 2021) when selecting model features and target variables. Our approach to problem formulation, model development, model operationalization and use within a clinical multi-disciplinary team were endorsed by the University of Glasgow public and patient involvement and engagement group. We undertook one-to-one interviews with patients with COPD. It was noted that patients were comfortable with model scores and insights being provided to clinicians on their care team, but that they did not expect or wish to see risk scores themselves. Our study participant information sheets were supplemented with additional written and video content hosted at our support website https://support.nhscopd.scot/dynamic-ai. Acceptability to patients (consent to trial recruitment) was selected as a co-primary objective along with technical feasibility of providing live AI model risk scores and safety of using these ML models for risk stratification and management optimization within COPD multi-disciplinary care team meetings as the primary objectives for the “DYNAMIC-AI” clinical investigation (NCT05914220). Prospective model performance and utility including the role of explainability and actionable insights are key secondary objectives.

### 3.2 Case study a: developing a 12-month mortality prediction model for patients with COPD using a large de-identified routine clinical dataset

#### 3.2.1 Problem formulation

For the first of these models a target variable of mortality was selected to get an overall view of the most high-risk patients in the cohort. A 12-month prediction window was selected to allow for a more long-term approach to risk reduction and anticipatory care planning to be considered at the point of inferencing. A windowing approach was used to allow for multiple observations per patient during model training. For example, if a patient had available data from 2015 to 2020, data from 2015 to 2019 would be used to predict mortality in 2020, and data from 2015 to 2018 would be used to predict mortality in 2019 and so on.

#### 3.2.2 The model development dataset

Following Local Privacy and Advisory Committee ethics review and approval, the West of Scotland Safe Haven (a collaboration between NHS GG&C and the Robertson Centre for biostatistics at the University of Glasgow) provided the data scientists in the technical team with a large deidentified dataset, in a cloud-based secure data environment. This dataset included healthcare data on hospital admissions, demographics, laboratory tests, prescriptions, and mortality for ~57,500 patients with COPD within NHS GG&C. The admissions data included admission events from 1997 up to 2022, the laboratory tests and prescriptions datasets included laboratory tests and prescriptions from 2008 up to 2022, and the mortality data included dates of death from 2010 up to 2022. This meant that a large dataset containing relevant healthcare data for the same population that the model was intended to run inference on was available for model development. Following discussions with Safe Haven it was determined that there no redactions of sensitive information from this dataset. At the relevant points in the model development process, approvals were obtained to share and discuss model and data artefacts within the MDT to ensure compliance with information governance standards.

#### 3.2.3 Data processing and feature engineering

The dataset was then split into a training cohort and an initial validation cohort, with patient data for 15% of the individuals in the dataset held back for the initial validation dataset. The first prediction year was set to 2013 to ensure data recency and to mitigate against data drift due to changes in hospital practices. Duplicate values and values outside of biologically plausible ranges were removed following guidance from the clinical team. As expected, the coverage for the laboratory tests included in the data varied significantly for different test types. Tests with <40% coverage at a population level were removed from the dataset to reduce the risk of introducing bias. For the remaining fields, both imputation of missing values using a harmonic mean and sparsity aware approaches were considered. Feature engineering was conducted to identify a full feature set using techniques such as categorical encoding and target encoding to deal with categorical and high cardinality features.

#### 3.2.4 Feature and algorithm selection

Clinically relevant features identified by the clinical team such as features related to recorded neutrophil: lymphocyte ratios, specified clinically relevant comorbidities, and inhaler medication history were combined with features identified during exploratory analysis by the data scientists in the technical team to create the full feature set. Logistic regression (Cox, 1958), support vector machine (Cortes and Vapnik, 1995), random forest (Breiman, 2001), and XGBoost (Chen and Guestrin, 2016) algorithm approaches were explored using implementations of these algorithms available through the scikit-learn Python package (Pedregosa et al., 2011). Deep learning methods were not explored as it was clear from collaborative discussions that approaches where the broad architecture of models could be easily understood were preferred. The XGBoost and random forest algorithms were further investigated based on the performance of these algorithms in the training data, the number of tunable hyperparameters available for these algorithms, and the established high performance of these algorithms with tabular datasets. The prospective models were then calibrated using Platt scaling and isotonic regression calibration approaches available via the scikit-learn Python package to try to increase the accuracy of the probability estimates provided by the models. The isotonic regression calibration approach was selected as it provided the best calibration between the distribution of the models predicted probabilities and the observed event occurrence rates. Ensuring model calibration was important so that the risk of mortality probabilities would be more meaningful and interpretable and so that as much as possible the individuals with a model score above the selected decision threshold would be the individuals at highest risk of mortality.

The prospective calibrated models were then evaluated on the initial validation dataset by the data scientists in the technical team and the results of this evaluation were shared with the clinical team. As this was a highly imbalanced problem, with mortality occurring within 12-months in around 8.3% of cases at the point of prediction, the area under the precision-recall curve was the primary metric used in performance evaluation. Confusion matrices were also created so that the utility and clinical feasibility of actioning the model at different decision thresholds could be discussed. Selecting a suitable decision threshold range was vital so that prospective models would identify a high proportion of those at the highest risk of mortality that it would also be feasible for the clinical team to review given that clinical capacity is limited. Despite the class imbalance, we did not use upsampling or downsampling techniques prior to model training as this reduced model calibration. Light hyperparameter tuning using the BayesSearchCV method from the scikit-optimize Python package (Head et al., 2020) was used to get a better indication of the performance of the XGBoost and random forest algorithms at this stage. A Bayesian optimization hyperparameter tuning approach was selected as this approach uses information on performant hyperparameter combinations identified during previous trials to inform the hyperparameter ranges explored in future searchers to get to a performant model in fewer runs (Snoek et al., 2012). Following this, a decision was made to focus entirely on XGBoost due to the higher performance of the XGBoost algorithms compared to the random forest algorithms, and the reduced risk of XGBoost algorithms overfitting to the data due to their regularization capabilities.

#### 3.2.5 Model explainability

**Figure 2:**
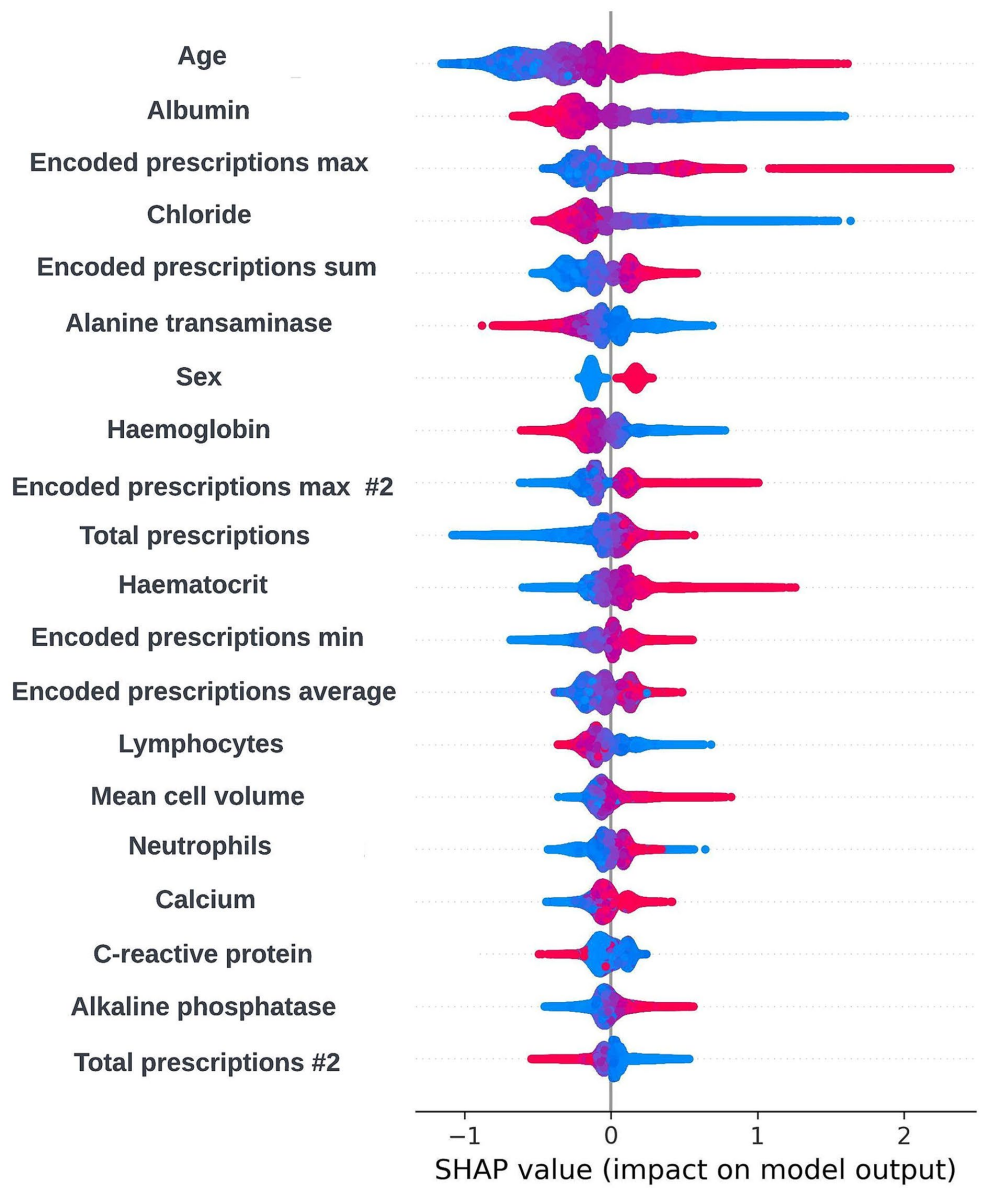
Global explainability view showing the SHAP values for the 20 most globally important features for a prospective 12-month mortality model; generalized feature names are used with numbered features representing features derived from the same data type. The SHAP values for each prediction are aggregated to get a global view of feature importance, with each dot representing an individual SHAP value. Positive SHAP values are attributed to features associated with mortality within 12-months and negative SHAP values are attributed to features associated with survival. The color of the dot represents the numerical value of the feature influencing the decision in the direction indicated by the SHAP value, with red dots representing high numerical values and blue dots representing low numerical values.

As XGBoost is not an inherently interpretable algorithm, frameworks for model agnostic explainability such as SHapley Additive exPlanations (SHAP) and local interpretable model-agnostic explanations (LIME) techniques were investigated (Lundberg and Lee, 2017; Ribeiro et al., 2016). SHAP, which uses a game-theoretic approach to quantify the impact each feature has on an individual prediction to get local level explainability, was selected as the preferred framework due to the tabular structure of the input data and the ability of the SHAP framework to produce a reliable global level view of explainability by combining the local level explainability estimates for each prediction. Global and local level SHAP plots were created using the “TreeExplainer” method from the SHAP Python package (Lundberg et al., 2020) and custom visualization techniques. Sessions were arranged between the clinical team and the data scientists in the technical team where these plots were discussed. In these sessions, it was determined that the workings of the model appeared to be biologically plausible and that there were not any obvious biases present. A global level SHAP plot in the style shared with the clinical team is shown in Figure 2.

#### 3.2.6 Model fairness

To consider the performance of the prospective models across different socioeconomic status (SES) groups, model performance was compared for individuals resident in postcodes in different deciles of the Scottish Index of Multiple deprivation (SIMD). The SIMD is the Scottish Government’s tool for measuring deprivation across all postcodes in Scotland which considers factors such as income, employment, health, housing, education, geographic access, and crime statistics (Scottish Government, 2023). To consider the performance of the model across different age groups and between sexes, the performances of the models were also compared between males and females, and people under and over the age of 65 years. The age, sex, and SIMD decile fields in the model development dataset all contained very low missing data levels. Across the different prediction windows in the training dataset, 55.34% of the training dataset were resident in postcode areas in the two most deprived deciles of the SIMD (1 and 2), 57.4% of the cohort were female, and 61.1% of the cohort were over the age of 65. These distributions are typical considering the burden of COPD at a population level in Scotland (Public Health Scotland, 2022). It was not possible to investigate performance by ethnicity using the available model development dataset, as there was no ethnicity data recorded for 16.3% of the cohort, and 82.3% of the cohort were recorded as white.

**Figure 3:**
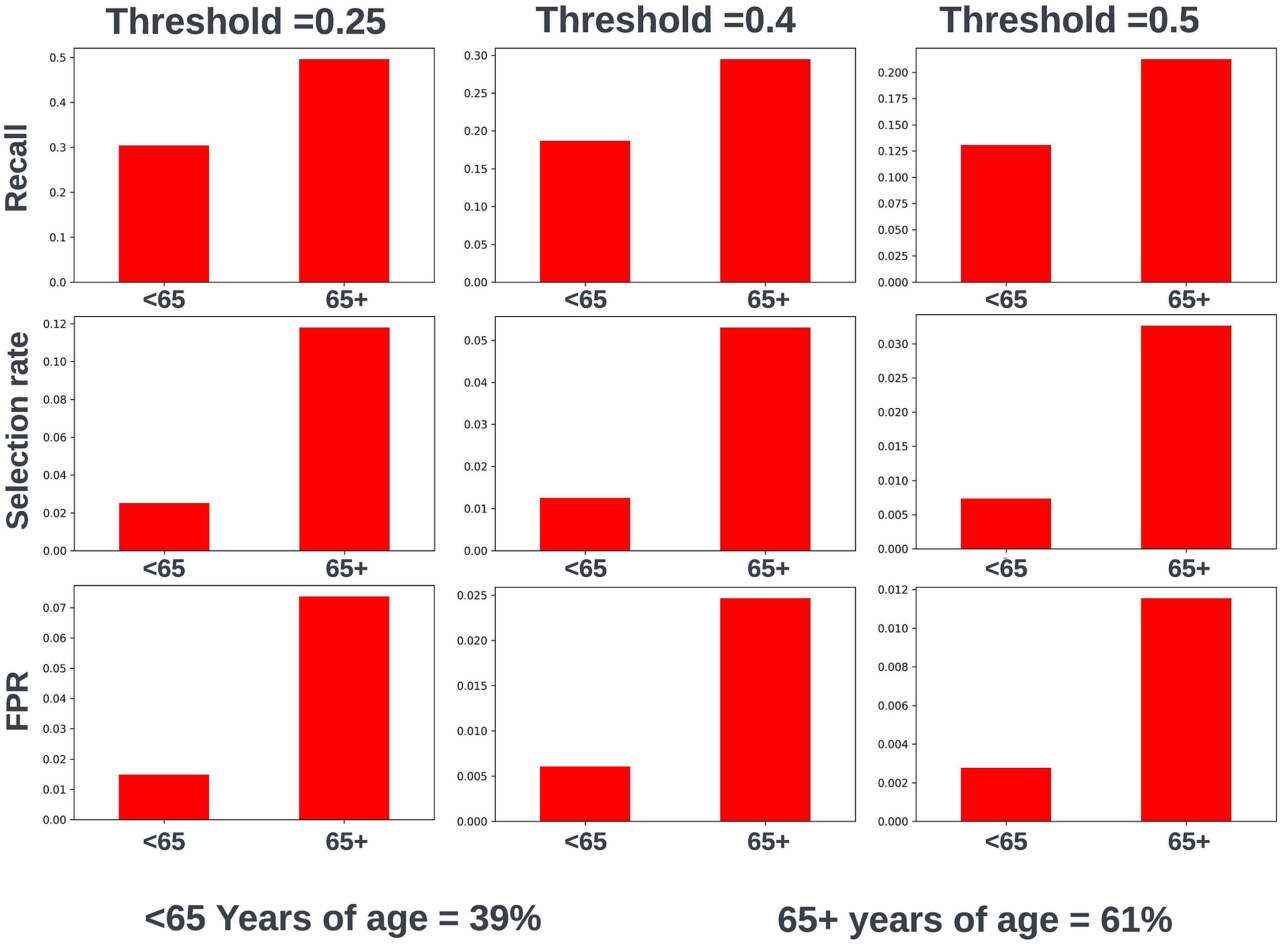
Recall, selection rate, and false positive rates for a prospective 12-month mortality model for people under and over 65 years of age in the validation dataset. These metrics are compared at various decision thresholds: 0.25, 0.4, and 0.5. The percentage of the cohort under and over the age of 65 years at the point of model prediction is also shown for context of the age group split within the cohort. FPR, false positive rate.

**Figure 4:**
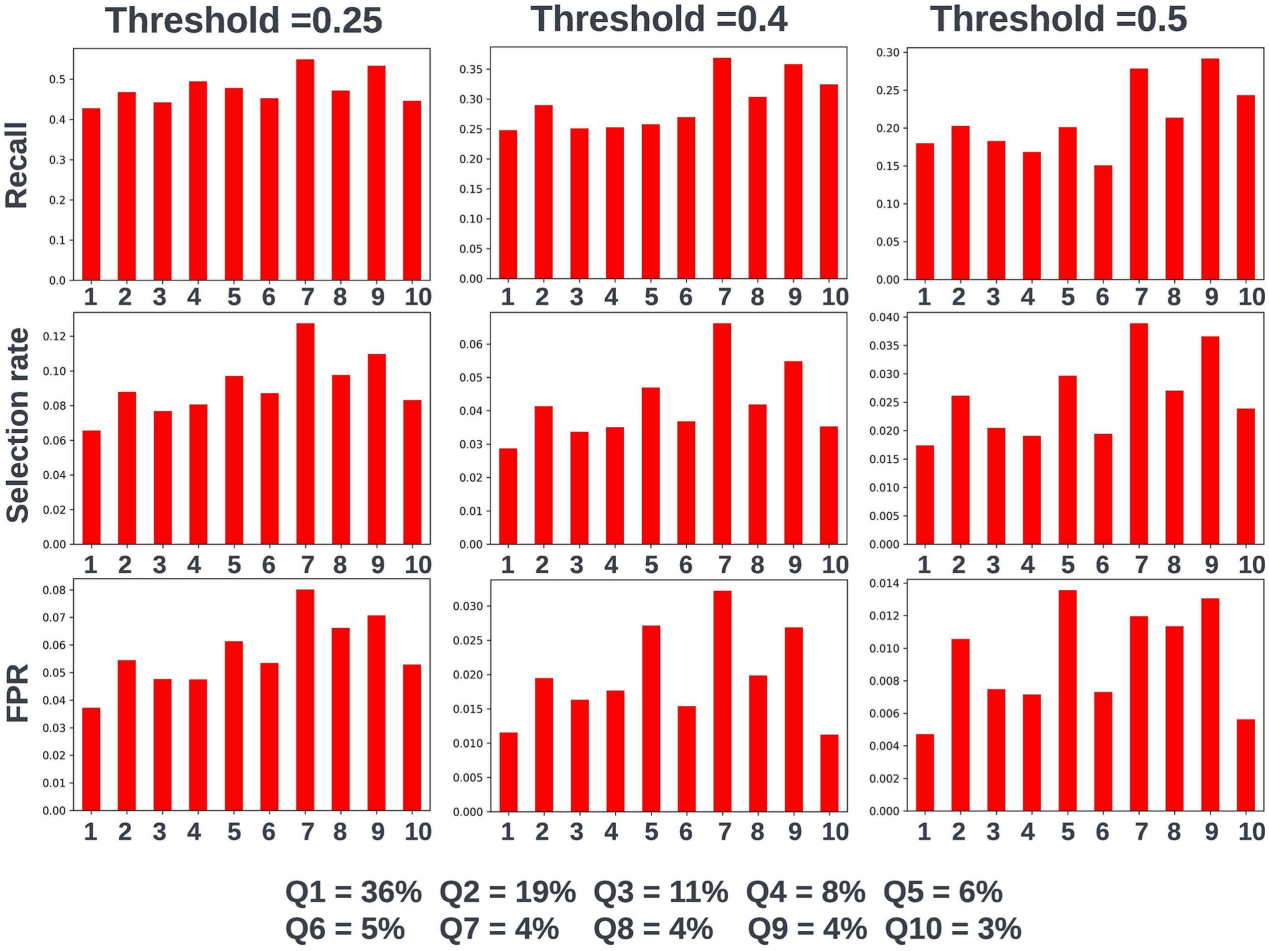
Recall, selection rate, and false positive rates for a prospective 12-month mortality model for people resident in postcodes in different deciles of the Scottish Index of Multiple Deprivation (SIMD) in the validation dataset. These metrics are compared at various decision thresholds: 0.25, 0.4, and 0.5. The percentage of the cohort resident in different deciles of the SIMD is also shown for context of the SIMD distribution within the cohort. The data shown for SIMD decile 1 represents predictions for individuals resident in the most deprived decile of the SIMD, the data shown for SIMD decile 10 represents predictions for individuals resident in the least deprived decile of the SIMD. FPR, false positive rate.

Fairness was investigated at various probability thresholds to fully understand how the model behaved across the different demographic groups. Analysis of model performance across the two age subgroups showed that the selection rate was substantially higher in those over the age of 65, across all investigated thresholds. Fewer selections in the younger group had the effect of a lower false positive rate at the trade-off of a lower recall (true positive rate) (Figure 3). A collaborative decision was made that it was acceptable to proceed with this difference at this stage, given that mortality risk increases significantly with age and therefore the model will logically select proportionally fewer younger people. Performance metrics were comparable between males and females at all investigated thresholds. When looking at performance metrics across SIMD deciles, it was determined that the models had a higher selection rate (and therefore increased recall but also a higher false positive rate) for individuals resident in the more affluent deciles. The disparity in recall was marginal at the low end of the trialed decision thresholds but increased at higher thresholds (Figure 4).

To mitigate any difference in performance metrics between subgroups, fairness aware loss functions were investigated. This involved using the Fairlearn wrapper for XGBoost (Weerts et al., 2023), which defines a custom loss function that looks to balance model performance across demographic groups of interest. However, this led to a global drop in performance meaning more opportunities for intervention would be missed across the cohort. Considering the improved fairness at lower decision thresholds for SIMD, the best option was determined to be focusing on the lower range of trialed thresholds for decision threshold selection, where marginal differences in recall between SIMD deciles were observed.

#### 3.2.7 Model selection and the model approval process

Following further hyperparameter tuning using k-fold cross validation Bayesian optimization, an XGBoost model was selected to bring forward for model approval. A K-fold cross validation approach was chosen to better understand how model performance would generalize to new data under different hyperparameter settings. A collaborative decision was made to allow missing laboratory test data due to the improved performance metrics of the algorithm without imputation and the ability of XGBoost to handle missing data well by grouping missing values at a decision node so that the loss function is minimized. Additionally, data explorations found that there were no systematic characteristic differences between patients with and without missing data other than a correlation between patients with more recent hospital attendances and data completeness for laboratory tests, but this was deemed acceptable as it did not introduce any undesirable biases into the model.

A model approval mechanism was created to ensure the clinical acceptability of any models approved for use in live clinical environment. The model approval process took place in two stages. The first stage of the process was a session involving the technical team, which was used to determine if all the low-level technical requirements for model operationalization were in place. In the second stage of the process, a model approval session took place involving the data scientist team and the clinical team, where the acceptability of the model for use in clinical care was assessed. The session was structured around a model approval document, which was created by the data scientists in the team and shared with the clinical team before the session. This document contained details of the training and validation process, a description of the model development dataset, a summary of the feature engineering process, a final feature set list, details of the algorithm used, a summary of the performance metrics of the selected model overall and across age, sex, and SIMD subgroups, and showed local and global model explainability views. Screenshots showing some of the core elements of the model reports used by our team are shown in Supplementary Figures 1, 2. The prospective model was accepted for approval without any changes required. Taking the stated fairness and clinical capacity factors into consideration, a decision threshold of 0.25 was selected as the number of patients brought forward at this threshold would be manageable for the COPD multidisciplinary care team to review and as the difference in model performance across SIMD groups was more marginal at this threshold.

**Figure 5:**
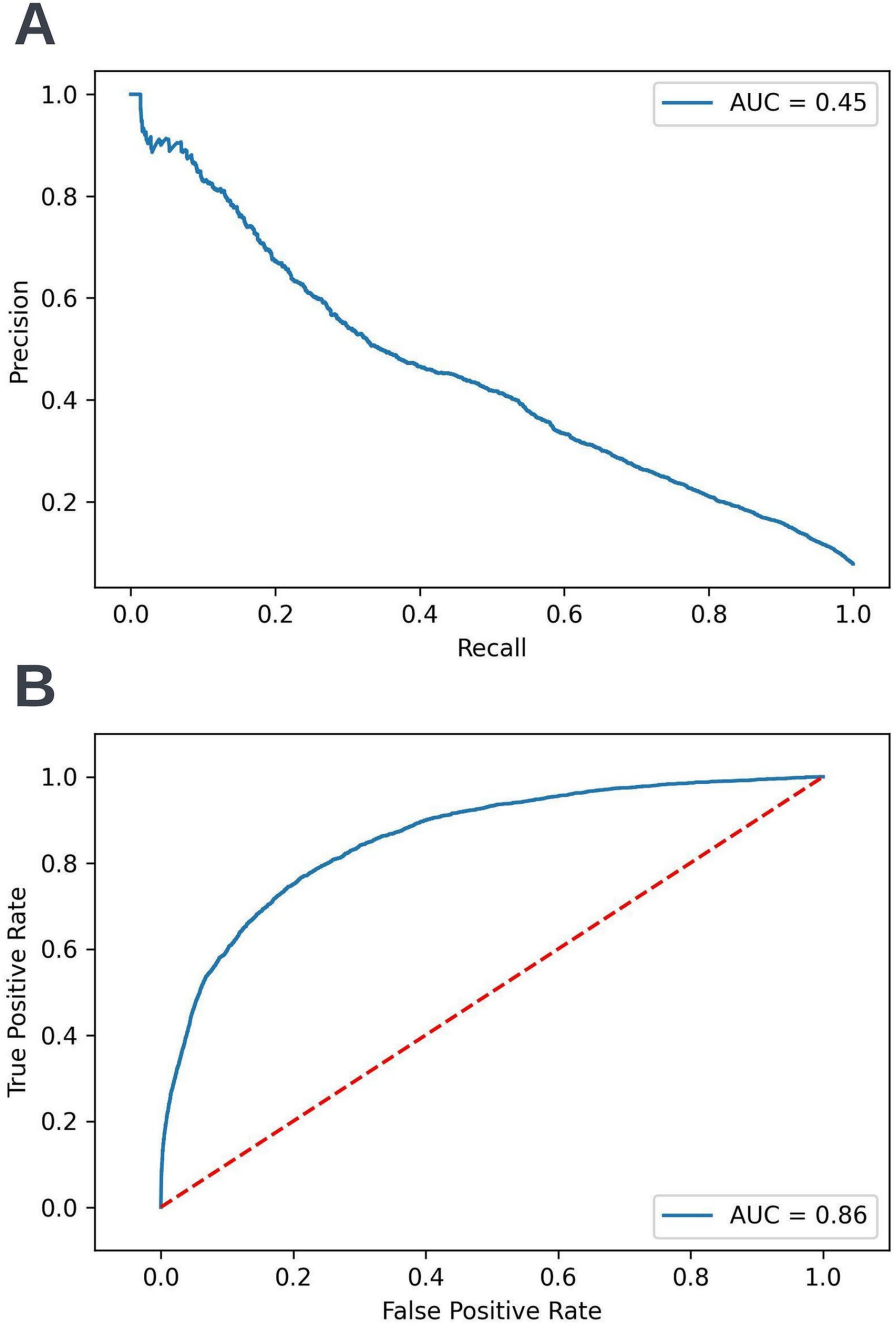
Precision-recall (A) and receiver operating characteristic curves (B) summarizing the performance of the selected 12-month mortality model in the validation dataset. In the precision-recall curve plot, the precision and recall of the selected model at different decision thresholds are shown (A). In the receiver operating characteristic curve plot, the true positive rate and the false positive rate at different decision thresholds are shown (B). AUC, area under curve.

**Figure 6:**
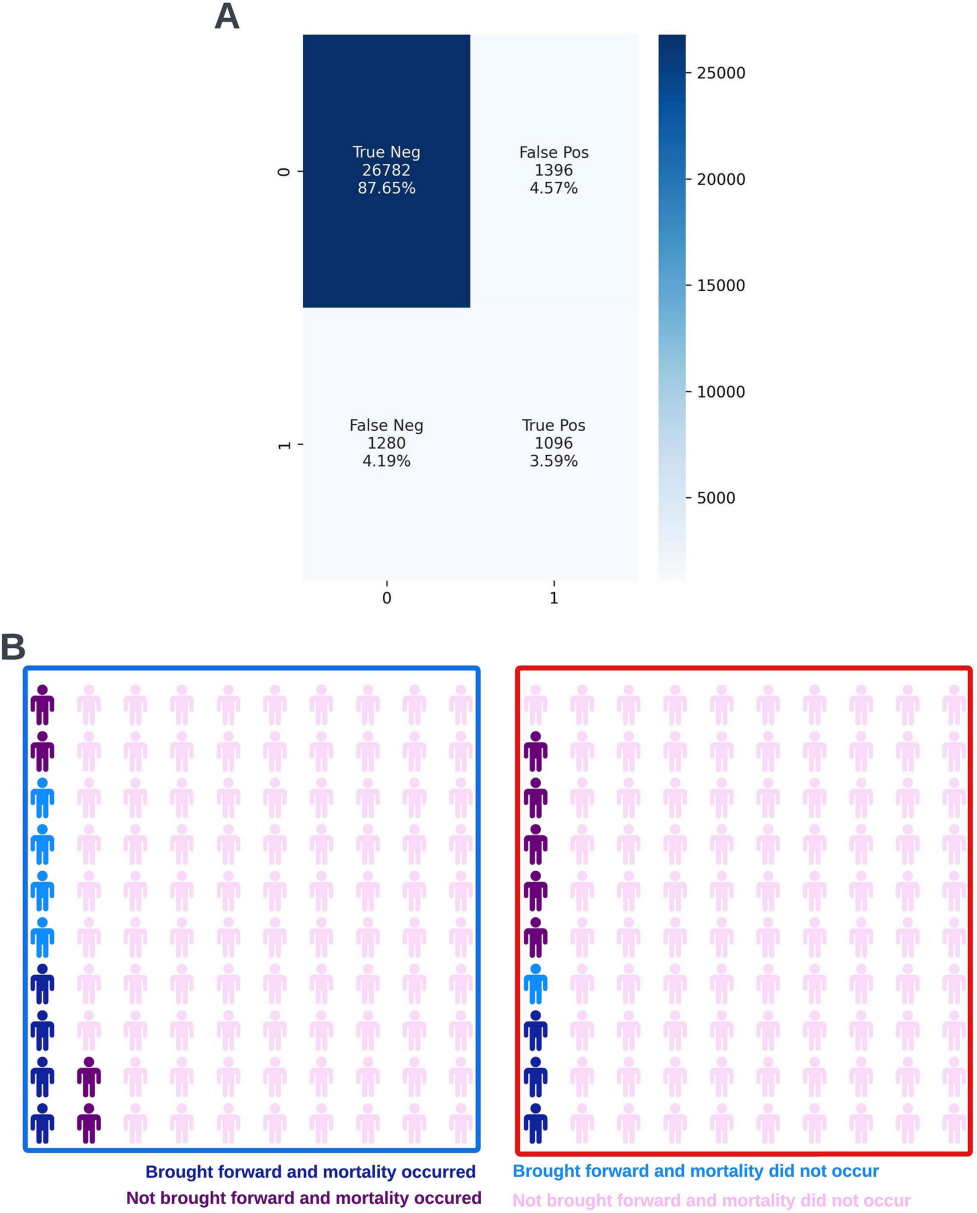
Confusion matrix (A) and illustrative infographic visuals (B) showing the workload manageability of the selected 12-month mortality model. The confusion matrix shows the number of true negatives, false positives, false negatives, and true positives when the selected model was run on the validation dataset at the selected decision threshold of 0.25. The illustrative infographic visuals are derived by normalizing the performance of the selected model in the validation set to 100 patients. The number of individuals that would be selected by the 12-month mortality model is shown at the selected decision threshold of 0.25 (left visual–blue box) and at a higher decision threshold of 0.4 (right visual–red box).

**Figure 7:**
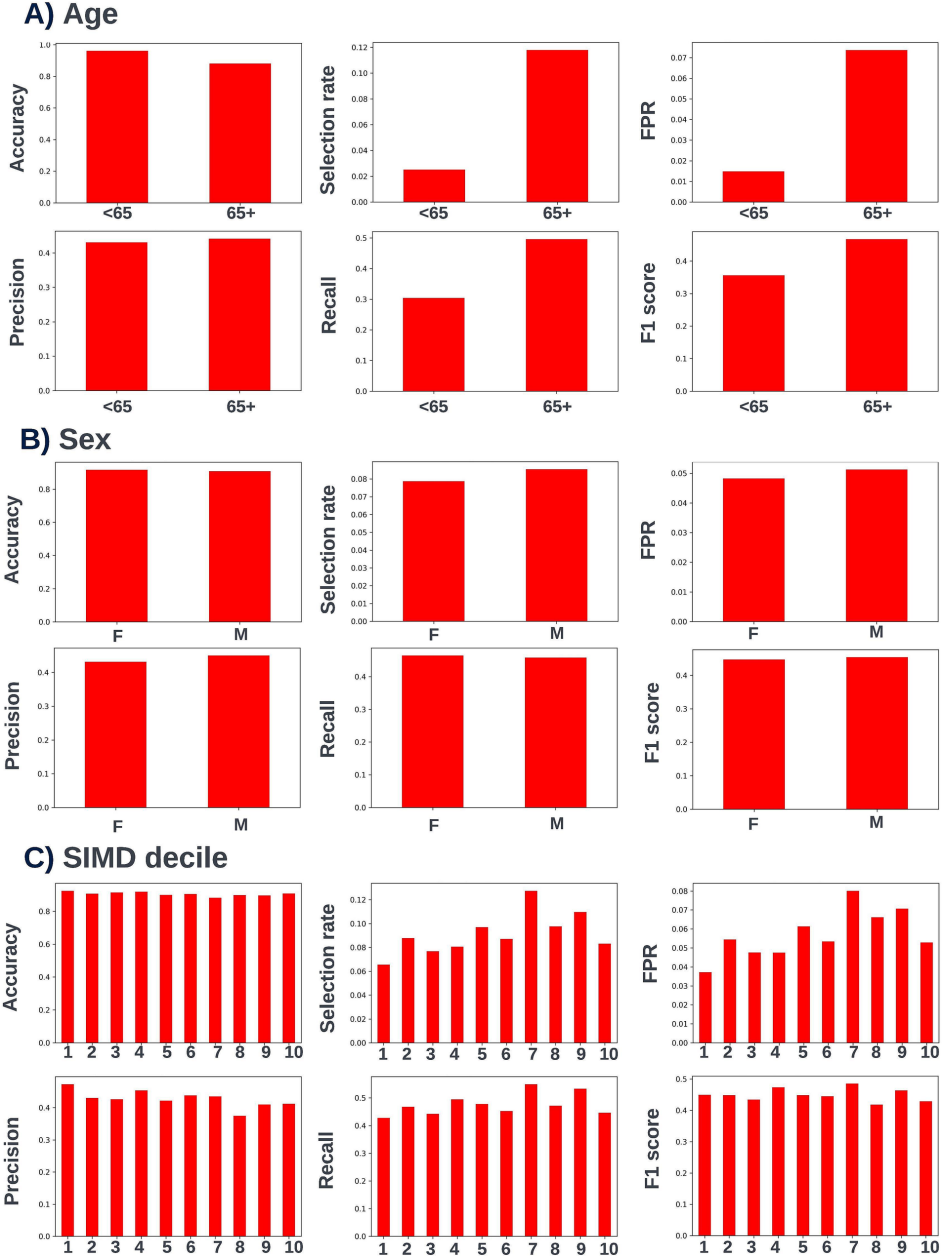
Performance metrics for the selected 12-month mortality model at the chosen decision threshold of 0.25 for individuals under and over the age of 65 years (A), for males and females (B), and for individuals resident in postcodes in different deciles of the SIMD (C). Accuracy, selection rate, false-positive rate, precision, recall, and F1 score metrics are shown. FPR, false positive rate.

A precision-recall curve and a receiver operating characteristic curve illustrating the performance of the selected model within the validation dataset are presented in Figure 5. The workload manageability of the model at the selected threshold of 0.25 is illustrated in Figure 6. A confusion matrix at the selected threshold and infographics showing the number of individuals out of 100 that would be correctly and incorrectly selected by the model at the selected threshold and at a higher threshold of 0.4 are shown based on the performance of the model in the validation dataset. A summary of the behavior of the final model across different age, sex, and SIMD subgroups at the selected threshold is shown in Figure 7.

#### 3.2.8 Defining routine clinical user needs and model operationalization

**Figure 8:**
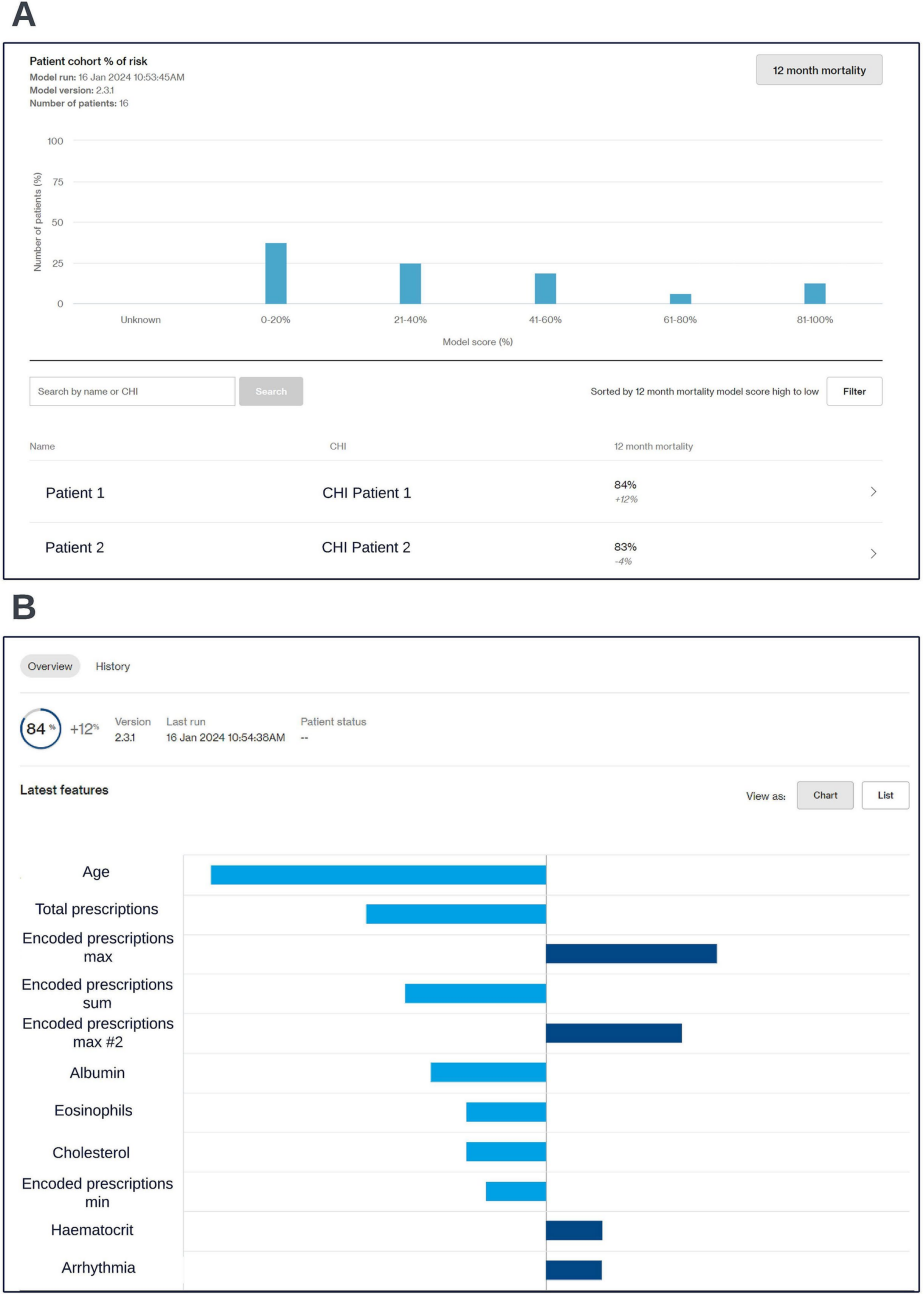
Screen capture views of the co-designed clinical dashboard. A cohort level view showing risk scores across the cohort (A) and an individual patient’s risk score and accompanying local explainability insights (B) are shown; generalized feature names are used with numbered features representing features derived from the same data type. The data shown is synthetic.

Preparation for operationalization occurred in parallel to model development. Codesign sessions were arranged to collaboratively define the best way to visualize model outputs. These sessions started prior to model selection and approval and involved the clinical and technical teams. The discussions centered around a dashboard capable of facilitating COPD multidisciplinary care team sessions, where high-risk patients would be reviewed. It was important that the dashboard could be viewed from any device, to reflect the diverse locations of these sessions. A website built using responsive design was chosen as the appropriate solution as it ensured portability and compatibility across different screen sizes. During codesign sessions, clinical participants highlighted the importance of providing an overview of the entire patient cohort and providing robust filtering and sorting capabilities to allow clinicians to easily identify high-risk patients. A significant focus was placed on displaying local explainability in a way that would enable clinicians to easily understand the factors driving risk for different individuals. Particular considerations were made to align the software with NHS workflows, including compliance with data privacy regulations and integration into existing clinical workflows. As part of the codesign we managed the risk of implementing the software into the NHS via a dedicated clinical risk log. All updates to the scope of model and data artefacts displayed in the live dashboard were approved by information governance. The resulting clinical dashboard is displayed in the screenshots below (Figure 8).

For the purpose of evaluation within a regulated clinical trial, individual participant consent for inference was required. Consent for participation was requested electronically for individual patient users of the Lenus COPD support service within NHS GG&C. Separate consent was sought from trial participants for the insights gained from the ML models to be used to inform their care. The data for consented individuals was processed into a format where it could be analyzed by the approved model via a data processing pipeline. Following this a machine learning pipeline was created using Azure ML studio to run the selected model on the processed data and return the results to the codesigned clinical dashboard. This allowed for model inferencing to be conducted for consented patients on an *ad-hoc* basis following requests from clinical users. Considering utility and clinical team availability to undertake multi-disciplinary review of outputs, model inferencing is currently conducted at approximately monthly intervals.

### 3.3 Case study B: developing a 90-day readmission prediction model for patients with COPD using a large de-identified routine clinical dataset

#### 3.3.1 Problem formulation

A target variable of readmission following discharge from a respiratory related hospital admission was selected to provide an alternative view of risk and to facilitate the clinical team undertaking data-driven triage of patients for additional inputs to try and reduce readmission rates specifically. A 90-day prediction window was selected so that more immediately at-risk patients could be identified. Given that this model was a readmission risk prediction model, the number of times each patient appeared in the processed training or validation data was dependent on the number of respiratory related hospital admissions they had had over the training window.

#### 3.3.2 The model development dataset

The dataset provided by the NHS GG&C Safe Haven for the 12-month mortality model was also used to develop the 90-day readmission model.

#### 3.3.3 Data processing and feature engineering

The subset of the full cohort in the model development dataset who had at least one respiratory related hospital admission were split into a training cohort and an initial validation cohort. To prevent data leakage individuals with multiple index dates could only appear in the training or validation dataset. 15% of patients with at least one respiratory related admission were included in the initial validation dataset. The same steps were taken to clean erroneous data and deal with missing values as were used with the 12-month mortality model formulation. Respiratory related admissions were identified using a series of ICD-10 codes provided by the clinical team. Index dates where mortality occurred within 90-days were not included in the training and validation datasets. Some of the features from the 12-month mortality model were retained in the full feature set for the 90-day readmission model, however in most cases feature formulations were adapted for the new model formulation. Additionally, no target encoded prescription features (which were used in the 12-month mortality model) could be created for the 90-day readmission prediction model formulation, as the current therapy prescription windows were on the same scale as the shorter prediction window making accurate target encoding not possible.

#### 3.3.4 Feature and algorithm selection

As before, features identified by the clinical team as likely having predictive and/or actionable utility were combined with features identified by the data scientists in the technical team during exploratory analysis to select an initial feature set. Logistic regression (Cox, 1958), support vector machine (Cortes and Vapnik, 1995), random forest (Breiman, 2001), and XGBoost (Chen and Guestrin, 2016) algorithm approaches were explored at first, using implementations of these algorithms available through the scikit-learn Python package (Pedregosa et al., 2011). XGBoost and random forest algorithms were further investigated based on the performance of these algorithms and the number of tunable hyperparameters available for these algorithms. These candidate algorithms were calibrated using an isotonic regression approach available through the scikit-learn Python package. The prospective calibrated models were then evaluated on the initial validation dataset. In the training dataset, 36% of admissions were followed by a subsequent admission within the following 90-days. Given this class balance, a combination of the area under precision-recall and receiver-operating characteristic curves were primarily used to evaluate model performance. Following light hyperparameter tuning a decision was made to focus entirely on XGBoost algorithms due to the higher performance of the XGBoost algorithms compared to the random forest algorithms in the validation dataset. Again, confusion matrices were created and shared with the clinical team to identify a range of clinically useful decision thresholds that would lead to a manageable workload for the team responsible for actioning the model outputs.

#### 3.3.5 Model explainability

**Figure 9:**
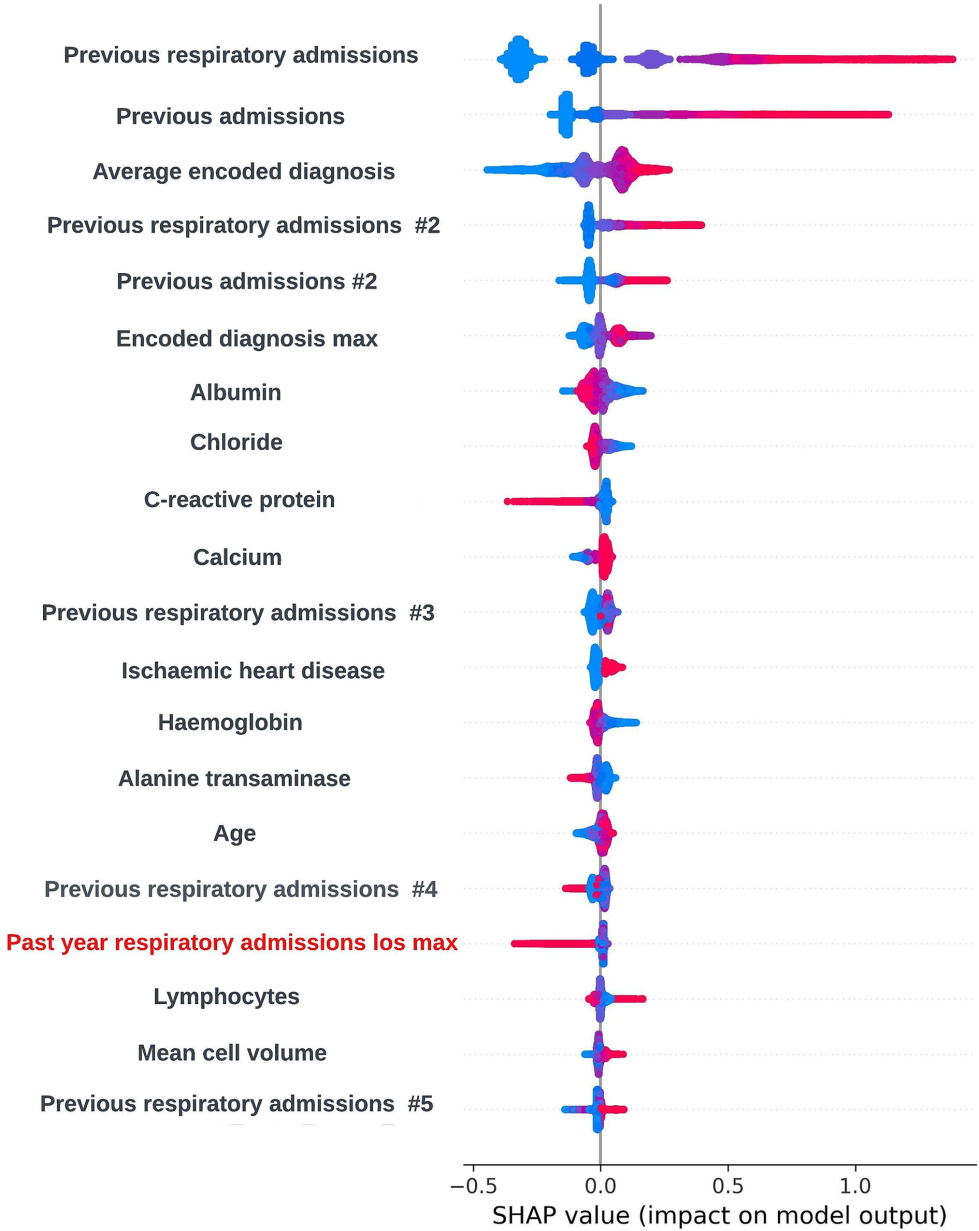
Global explainability view showing the SHAP values for the 20 most globally important features for a biased 90-day readmission model; generalized feature names are used with numbered features representing features derived from the same data type. The SHAP values for each prediction are aggregated to get a global view of feature importance, with each dot representing an individual SHAP value. Positive SHAP values are attributed to features associated with readmission occurring within 90-days of the index admission start date and negative SHAP values are attributed to features associated with no readmission occurring within 90-days of the index admission start date. The color of the dot represents the numerical value of the feature influencing the decision in the direction indicated by the SHAP value, with red dots representing high numerical values and blue dots representing low numerical values. The label for the biased feature that incorrectly showed that a higher maximum length of hospital stay in the previous 12-months was associated with lower readmission risk is shown in red.

**Figure 10:**
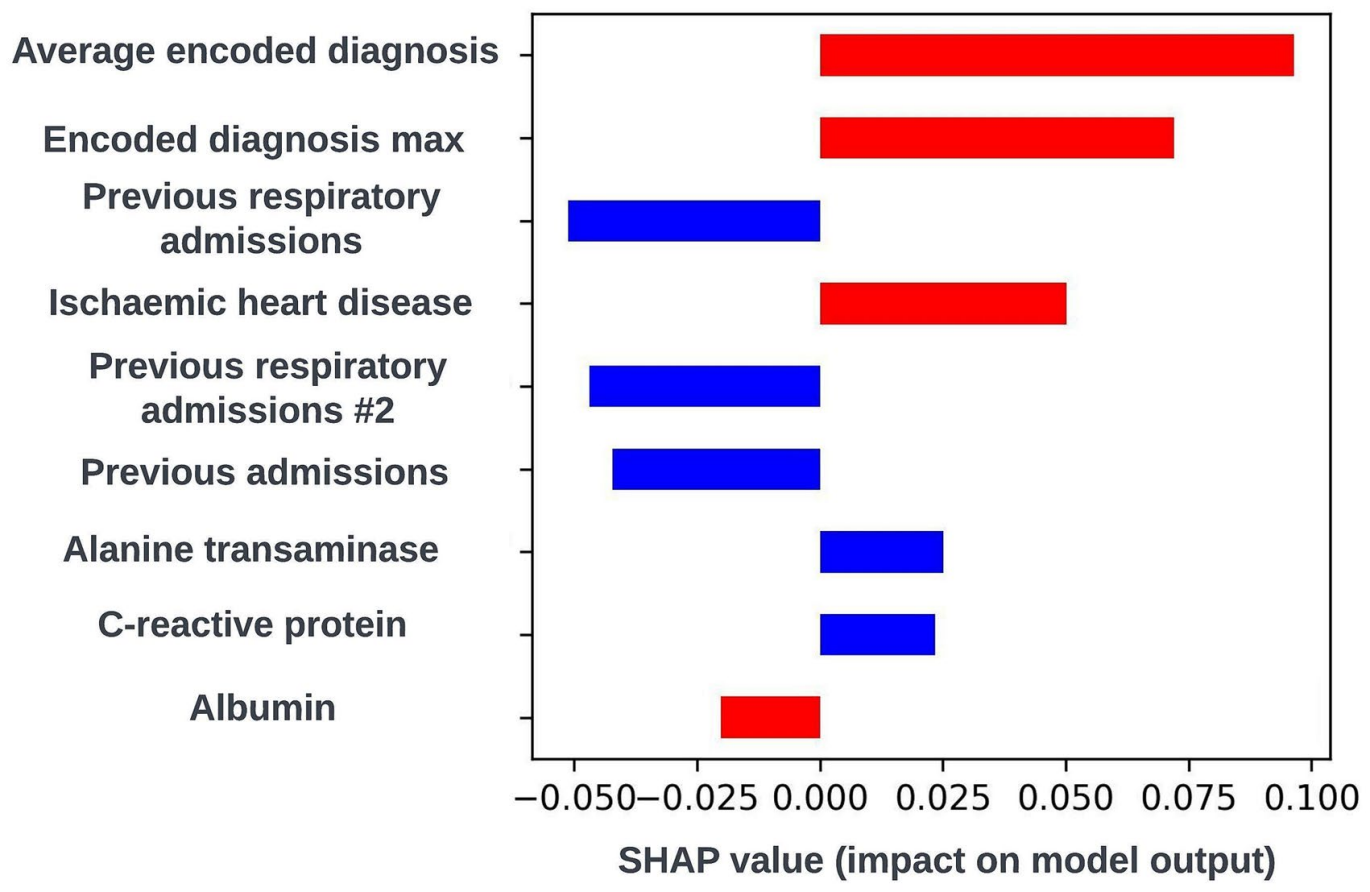
Local explainability view for a biased 90-day readmission model showing the seven features with the greatest SHAP values for an illustrative prediction for an individual patient; generalized feature names are used with numbered features representing features derived from the same data type. Positive SHAP values are attributed to features associated with readmission occurring within 90-days of the index admission start date and negative SHAP values are attributed to features associated with no readmission occurring within 90-days of the index admission start date. The color of the bar represents the numerical value of the feature influencing the decision in the direction indicated by the SHAP value, with red bars representing high numerical values and blue bars representing low numerical values.

The SHAP framework (Lundberg and Lee, 2017) was applied to prospective models using the “TreeExplainer” method from the SHAP Python package (Lundberg et al., 2020) and SHAP plots were discussed with the clinical team during dedicated sessions to identify any biases and ensure the biological plausibility of the workings of the model. During a model explainability session for an exploratory formulation of this model, it was observed that an increase in the maximum length of time an individual had spent in hospital during respiratory related admissions over the previous 12-months was associated with a lower risk of readmission within 90-days. This was deemed to be unexpected by the clinical team, and so the data science team investigated if any potential biases could be causing this effect. It transpired that this was a result of an oversight in this formulation whereby the 90-day prediction window began at the admission date as opposed to the discharge date for the index admission event. This resulted in the model learning that people who were still in hospital after a hospital admission of 90-days or longer would not readmit. The formulation was then adjusted to resolve this issue. However, this incident exemplifies the importance of this process. If explainability views were not available, this bias in model formulation may not have been identified and the model could fail to identify a subgroup of patients likely to be at high-risk of readmission. A global SHAP plot for a model from this point in model development which highlights the feature described above is shown in Figure 9. An illustrative custom local level SHAP plot in the style used by our team to look at key risk factors for individual patients at this point in model development is also presented in Figure 10.

#### 3.3.6 Model fairness

**Figure 11:**
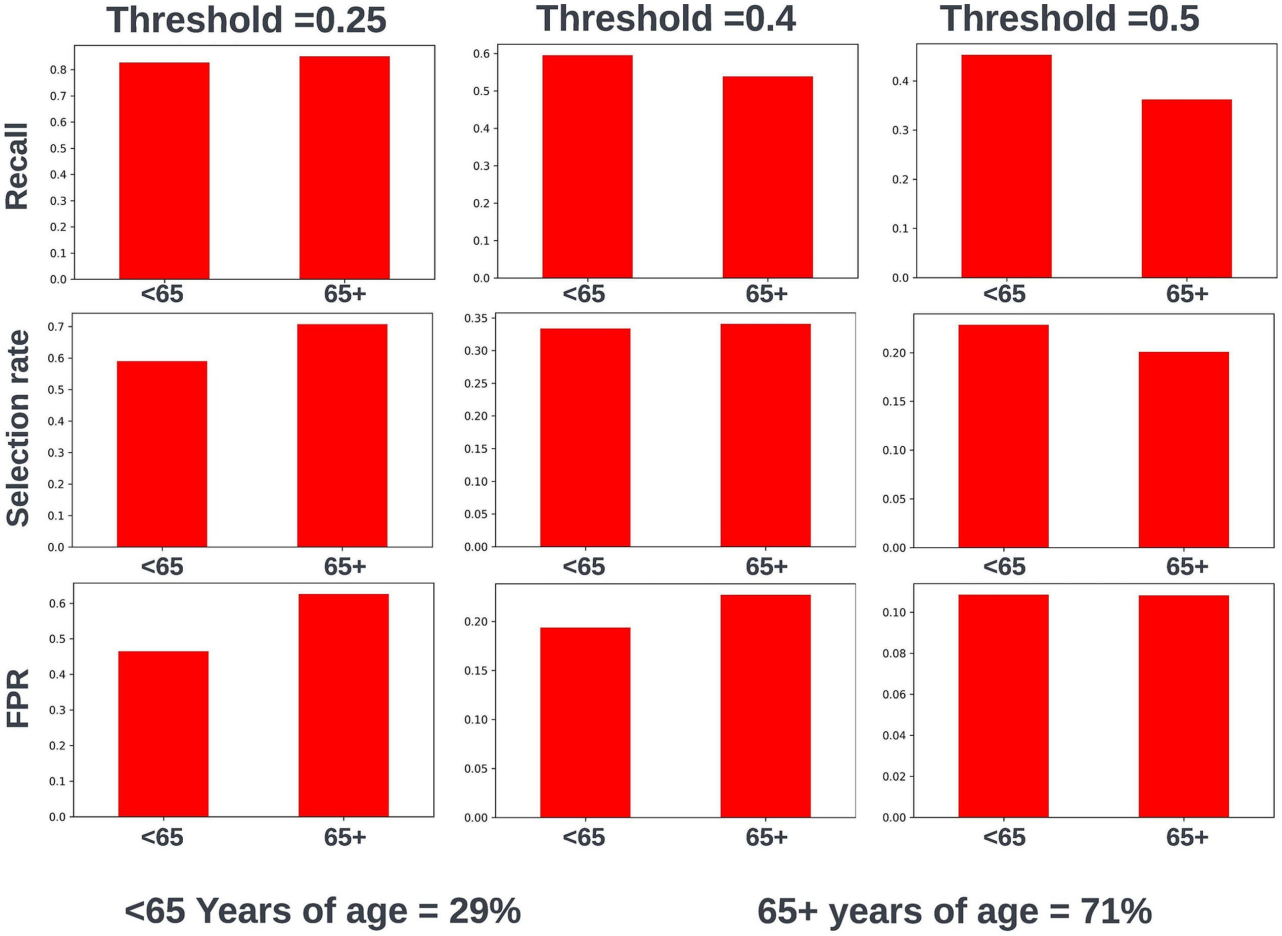
Recall, selection rate, and false-positive rate for a prospective 90-day readmission model for people under and over 65 years of age in the validation dataset. These metrics are compared at a range of decision thresholds: 0.25, 0.4, and 0.5. The percentage of the cohort under and over the age of 65 at the point of prediction is also shown for context of the age group split within the cohort. FPR, false positive rate.

**Figure 12:**
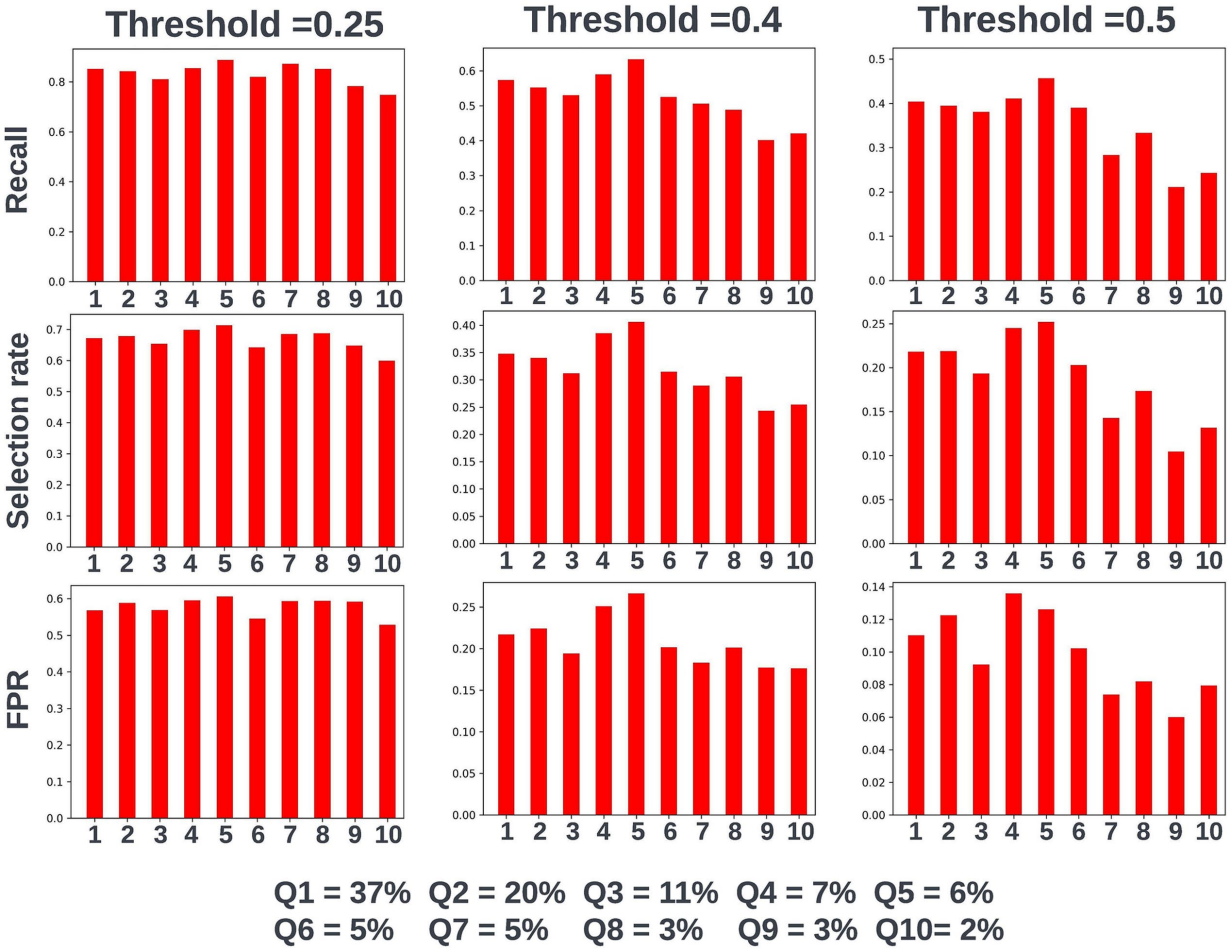
Recall, selection rate, and false-positive rate for a prospective 90-day readmission model for individuals resident in postcodes in different deciles of the SIMD in the validation dataset. These metrics are compared at a range of decision thresholds: 0.25, 0.4, and 0.5. The percentage of the cohort resident in different deciles of the SIMD is also shown for context of the SIMD distribution within the cohort. The data shown for SIMD decile 1 represents predictions for individuals resident in the most deprived decile of the SIMD, the data shown for SIMD decile 10 represents predictions for individuals resident in the least deprived decile of the SIMD. FPR = false positive rate.

Again, fairness was assessed across age, SIMD, and sex demographic subgroups. Across the different prediction windows in the training dataset, 57.22% of the cohort were resident in postcode areas in the most deprived two deciles of the SIMD, 59% of the cohort were female, and 61.1% of the cohort were over the age of 65 years. Analysis of model performance at different decision thresholds showed that at lower thresholds, recall was equivalent between those under and over the age of 65, whilst the selection rate and false-positive rate were higher in those over the age of 65. However, as the selected decision threshold increased, recall, and subsequently selection rate became comparatively higher in those under 65 (Figure 11). A similar effect was observed when looking at performance across the different SIMD decile subgroups, with a growing gap seen in model performance and selection rate for individuals resident in postcode areas in the more deprived deciles (higher selection rate and recall) and the more affluent deciles (lower selection rate and recall) as the decision threshold increased (Figure 12). Performance metrics were comparable between males and females at all investigated thresholds.

#### 3.3.7 Model selection and the model approval process

**Figure 13:**
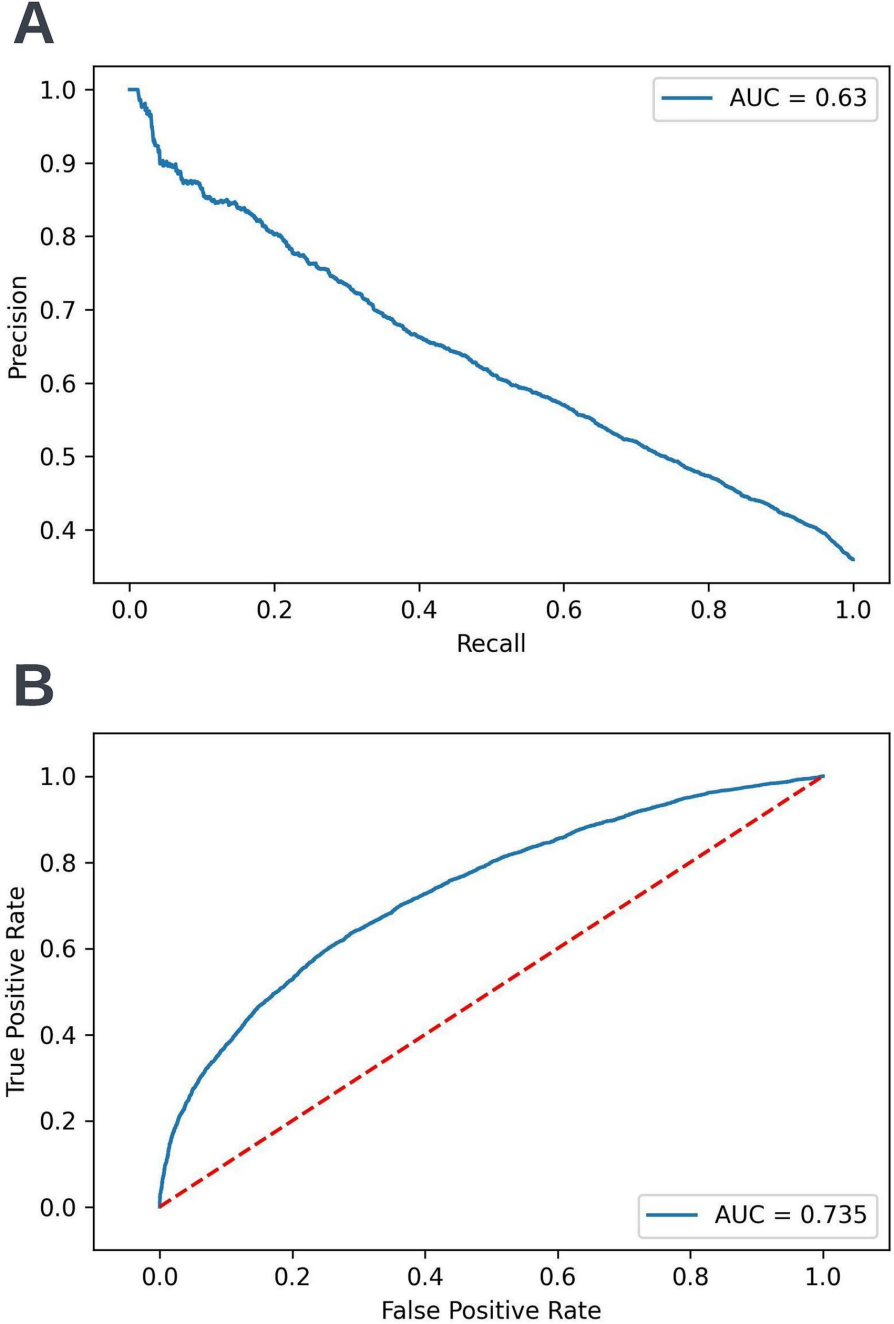
Precision-recall (A) and receiver operating characteristic curves (B) summarizing the performance of the selected 90-day readmission model in the validation dataset. In the precision-recall curve plot, the precision and recall of the selected model are shown at different decision thresholds (A). In the receiver operating characteristic curve plot, the true positive rate and the false positive rate at different decision thresholds are shown (B). AUC, area under curve.

**Figure 14:**
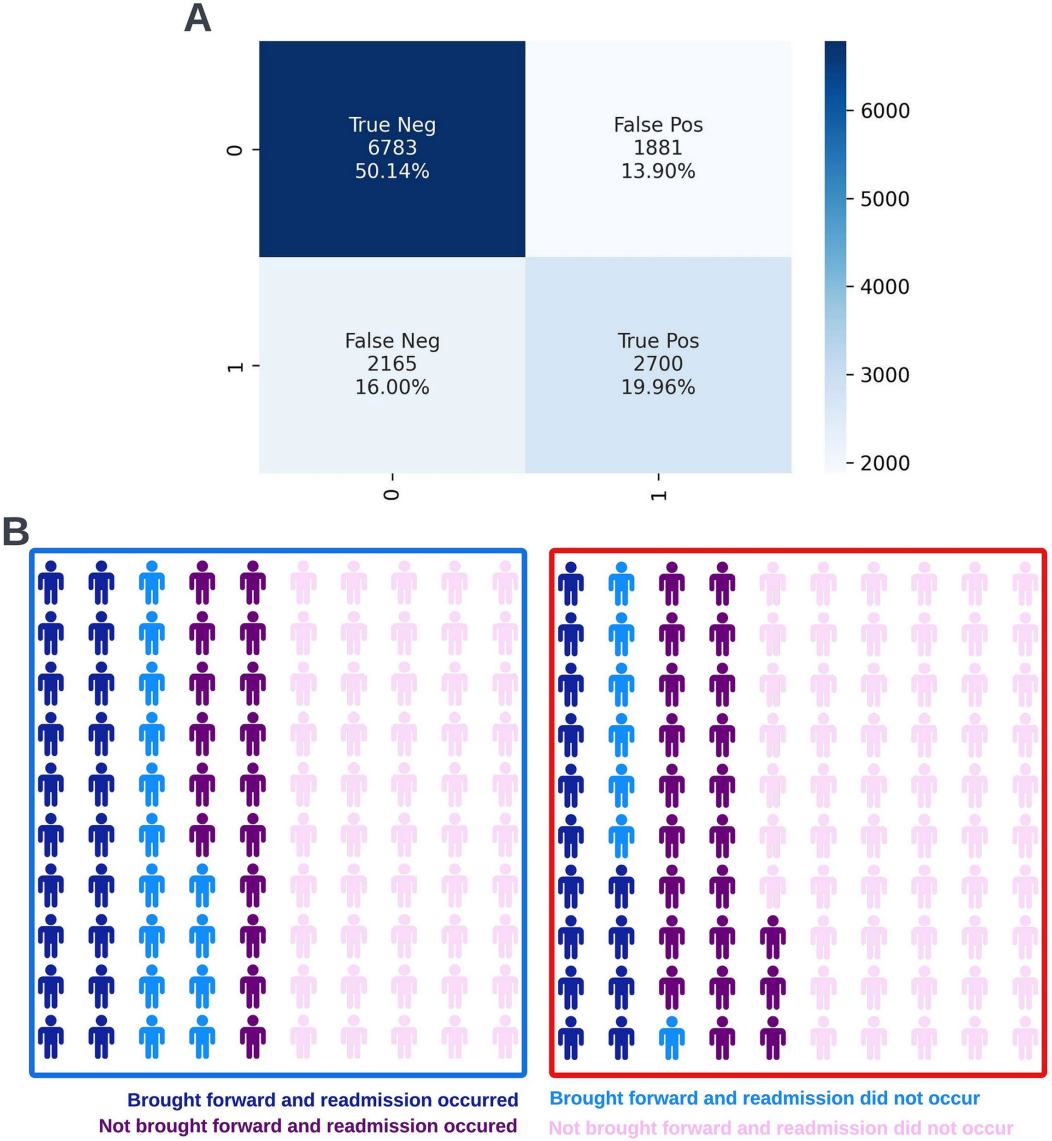
Confusion matrix (A) and illustrative infographic visuals (B) showing the workload manageability of the selected 90-day readmission model. The confusion matrix shows the number of true negatives, false positives, false negatives, and true positives when the selected model was run on the validation dataset at the selected decision threshold of 0.4. The illustrative infographic visuals are based on normalizing the performance of the selected model in the validation set to 100 patients. The number of individuals that would be brought forward by the 90-day readmission model is shown at the selected decision threshold of 0.4 (left visual–blue box) and at a higher decision threshold of 0.5 (right visual–red box).

**Figure 15:**
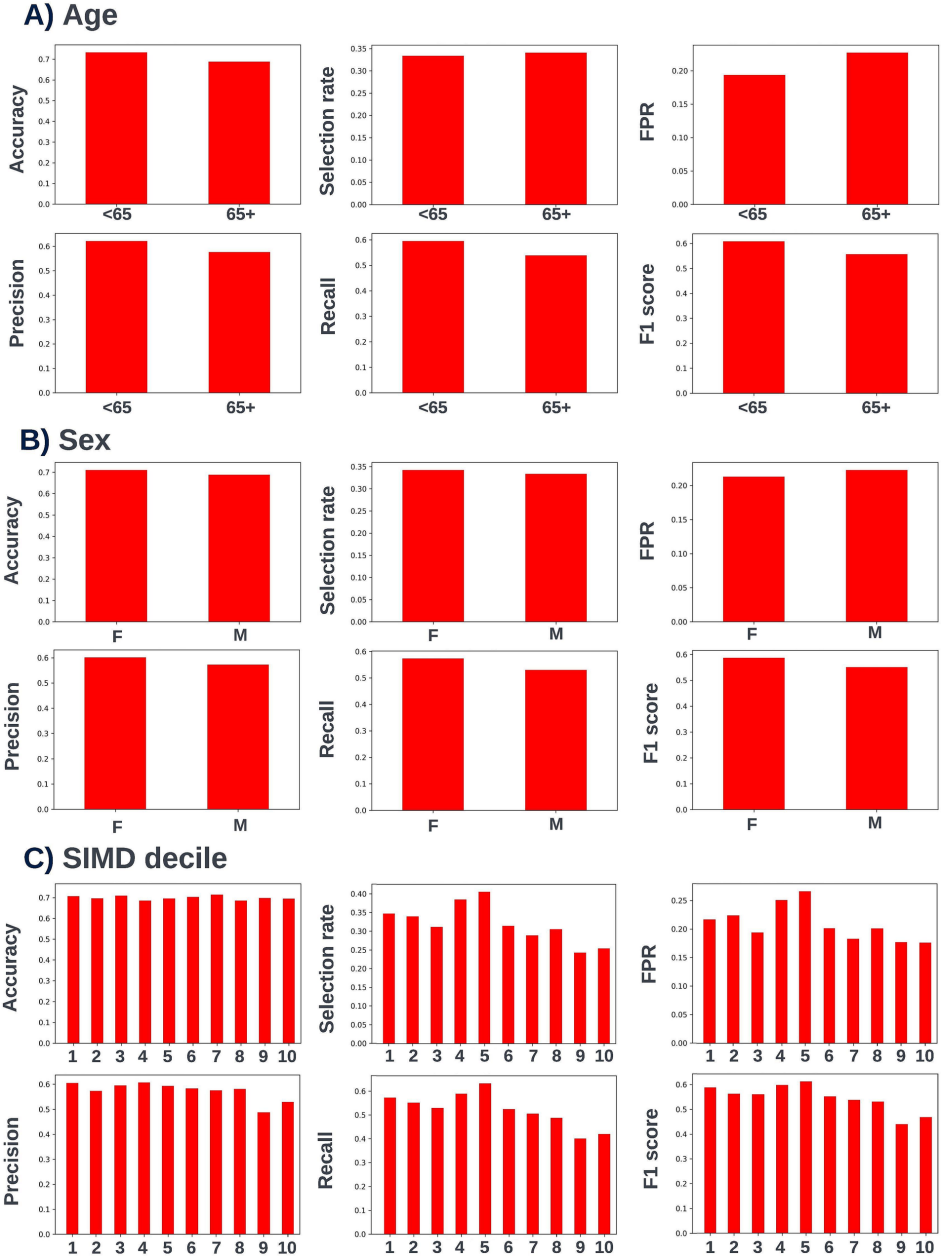
Performance metrics for the selected 90-day readmission model at the chosen decision threshold of 0.4 for individuals under and over the age of 65 years (A), for males and females (B), and for individuals resident in postcodes in different deciles of the SIMD (C). Accuracy, selection rate, false-positive rate, precision, recall, and F1 score metrics are shown. FPR, false positive rate.

Following hyperparameter tuning to increase the performance of the candidate models using k-fold cross validation Bayesian optimization with the BayesSearchCV method from the scikit-optimize Python package (Head et al., 2020), an XGBoost model was selected for model approval. Again, a sparsity aware approach was chosen due to the increased performance of the model when missing data was included. This prospective model underwent the two-stage model approval process and was approved for use. A decision threshold of 0.4 was selected based on discussions around the clinically useful threshold range and as more marginal differences in recall between age and SIMD subgroups were observed at this threshold. The precision-recall and receiver operating characteristic curves illustrating the performance of the selected model within the validation set are presented in Figure 13. The workload manageability of the model at the selected threshold of 0.4 is illustrated in Figure 14. A confusion matrix at the selected threshold and infographics showing the number of individuals out of 100 that would be correctly and incorrectly selected by the model at the selected threshold and at a higher threshold of 0.5 are shown, based on the performance of the model in the validation dataset. A summary of the behavior of the final model across different age, sex, and SIMD subgroups at the selected threshold is shown in Figure 15.

#### 3.3.8 Defining routine clinical user needs, model operationalization

It was determined through collaborative discussions between the technical team and the clinical team that the model risk scores and accompanying explainability views should be added to the existing clinical dashboard to provide clinical users with multiple views of risk for the patient population in one place. This update required approval from information governance. Consent for the insights gained from model inferencing to be used to inform the care of the trial participants was covered by the same process as the 12-month mortality model. A data processing pipeline was created to transform the EHR data for the relevant consented patients (those with a discharge date for a respiratory related admission within the previous 90-days) into the required format for inferencing. An Azure ML studio pipeline was created to run the model on the processed data and return the risk scores and explainability views for each of these patients to the clinical dashboard. Model inferencing for the 90-day readmission model is currently conducted on an *ad-hoc* basis as requested by the clinical team.

## 4 Discussion

A lack of resources and means of oversight of the growing number of individuals affected by long-term conditions means the current care model for these conditions is often disjointed and reactive, resulting in poorer patient outcomes and avoidable hospital admissions. Electronic health record (EHR) data provides a rich source of information related to patient’s medical histories that does not require additional data collection or tests. However, this data is typically presented in an unaggregated row level format and is accessed across different locations, making it difficult to harness this data to assist in the management of patient cohorts given the time pressures on clinical teams. In recent years, there has been significant interest in training machine learning models from this data to gain insights to inform the management of patient cohorts (Habehh and Gohel, 2021; Sauer et al., 2022). However, most of the investigation into this has been exploratory work without clinical involvement and there has been limited translation of this work into tools used in clinical practice (Bastian et al., 2022). The objectives of this paper were to outline a detailed collaborative workflow based on the experience of our multidisciplinary team that can be followed to promote the use of ML tools in live clinical environments. The development and operationalization of two of the models developed by our team for clinical decision support for COPD in NHS GG&C which informed this framework are also described in two case studies to give real-world context to the considerations that have to be made at each stage of this process and highlight the lessons that were learned through this work. Based on the interim analysis of the primary outputs of the trial which demonstrate the safety, feasibility and acceptability of the use of ML models in live clinical practice (Taylor, 2024), we expect that the clinical team in NHS GG&C will continue to use the models in routine clinical practice following the completion of the DYNAMIC AI trial in January 2025.

There are several key points to take away from this framework and the included case studies. Firstly, it is crucial to have involvement from a clinical team with extensive knowledge of the target condition and experience of the current care pathway for that condition at every stage of the process. Clinical involvement will ensure that project outputs have maximal clinical utility, provide context to the nuances of the EHR data, and provide a clinical perspective to tackle wider issues related to the use of ML in healthcare such as model interpretability and fairness. Secondly, decisions made at even the earliest stages can drastically impact the feasibility and ease of operationalizing and adopting models within existing care pathways and so it is crucial to consider the implication of any decisions made on this end goal. Thirdly, in our view determining an appropriate method for displaying model insights and accompanying explainability views should be considered as important as identifying performant models. Clinical teams are under significant time pressures and providing a way of sharing insights that is intuitive and accessible, whilst providing maximum information is key. Fourthly, there should be a documented and traceable process for approving any models that may be used in live clinical practice. This process should record detailed information about the performance, fairness, and explainability of any models put forward for approval, as well as a description of the algorithm used, and an overview of the training process. Based on this information, the clinical team should assess the model’s acceptability for use in a live clinical environment, with any subsequent iterations undergoing this same process. Lastly, although the required work is achievable, there can be significant delays at various stages of this process (particularly around governance) and significant time and resources should be allocated to these projects.

The workflow described in this paper draws heavily from the work processes carried out by our multidisciplinary team in NHS GG&C as described in the two case studies. However, following the learnings from this project we have adjusted our own working processes and would recommend a distinct approach to that carried out in the presented case studies in a few key areas. For example, fairness analysis was only conducted for XGBoost algorithms for both models in NHS GG&C as investigation into other algorithms was stopped earlier in the process. However, we would recommend exploring fairness across multiple different algorithms by tracking model performance metrics of interest across different demographic groups of interest at different thresholds to ensure model fairness considerations are incorporated into algorithm selection. Additionally, we did not include SIMD decile as a model feature due to concerns that the model would capture confounding societal effects. Conversely, more recent approaches have started to use deprivation indexes as model features and suggest that it is fairer to do this as this avoids introducing an omitted variable bias (Li et al., 2022). As a result, we recommend including all investigated fairness parameters as model features.

There were also a few constraints that impacted the work carried out during this project. For example, we would have liked to have presented data drift and model performance monitoring tools in the first implementation of the clinician dashboard. However, this wasn’t possible as the methods for acquiring these measures within the inferencing infrastructure rely on training data being stored in the same environment as the inference data, which was not possible with our project due to governance restrictions. This is something that we would like to explore in another project. As another example, fairness by ethnic group could not be looked at robustly for either of the models due to the lack of recorded representation of people from non-white ethnic groups within the model development dataset. However, we strongly recommend looking at fairness by ethnic group if this is possible using the training dataset available given that bias in performance by ethnic group has been observed in models developed from EHR data (Gianfrancesco et al., 2018). Further work will be required to explore fairness by ethnic group in another dataset if these models are to be scaled or used in other locations/populations.

This workflow framework focused on models deriving features from the structured data contained within EHRs and supervised machine learning approaches to avoid overcomplicating the outlined workflow stages. Models deriving features from unstructured data (such as clinical notes within EHRs) would require an entirely different approach to dataset exploration, data processing, feature generation, and deriving and presenting model explainability. Separately, with an unsupervised approach (such as developing clustering algorithms using EHR data) the methods for assessing model performance, deriving model explainability, and exploring model fairness would be very different to the approaches described in the case studies. However, the need to involve clinical and technical teams from the start of the project to ensure the explainability, fairness, actionability, and clinical utility of models, as well as the need for a traceable model approval process would equally apply to these projects.

The case studies presented in this paper look at two models used to inform COPD care in a secondary care setting. Whilst the core framework steps are applicable across all chronic disease indications and care settings, the exact approach applied will be influenced by the long-term condition that is the focus of the project and the priorities of patients with that condition and the intended care setting in which the model will be used. For example, individuals with COPD experience particularly high readmission rates and report that exacerbations are the most disruptive aspect of their condition (Alqahtani et al., 2021). Avoiding these events is therefore a key care priority and so our MDT developed a respiratory related readmission risk model to be run at the point of discharge from a respiratory related hospital admission. Separately, the use case of the models to support specialist clinical MDT care meetings meant that a high level of detail was included in model explainability outputs. Projects focusing on implementing models in other care settings should tailor the ways of presenting model derived insights and accompanying explainability to suit the needs of clinical care teams in these settings where those interacting with models may not be specialists on the disease indication the model has been developed for.

## 5 Conclusion

The adoption of machine learning models in clinical care could provide a more predictive, proactive, and personalized approach to the management of long-term conditions. However, there are only a limited number of these tools being used in clinical practice and there is a need to generate real-world evidence of the impact of these models. In this paper, we have defined a workflow framework for the development and operationalization of machine learning models from EHR data to assist with the management of long-term conditions and presented two case studies to provide context to how this approach was informed. Following this framework can push forward progression in this space, leading to a larger number of EHR derived machine learning models being operationalized, adopted, and evaluated for clinical and cost-effectiveness.

## Data Availability

The data analyzed in this study is subject to the following licenses/restrictions: the dataset used for the development of the 12-month mortality and 90-day readmission risk models presented in the case studies in this paper are available for access via NHS GG&C Safe Haven. Applications for de-identified data access would be reviewed by the NHS GG&C Local Privacy and Advisory Committee and might incur data access fees. Requests to access these datasets should be directed to safehaven@ggc.scot.nhs.uk.
