# Supplementary material for: Supporting long-term condition management: a workflow framework for the co-development and operationalization of machine learning models using electronic health record data insights
Source: Front Artif Intell. 2024 Nov 12;7:1458508. doi: 10.3389/frai.2024.1458508 (PMC11588744; doi:10.3389/frai.2024.1458508)
Supplement: Supplementary file 1 [file Image_1.pdf]

Supplementary Material

1 Supplementary Figures

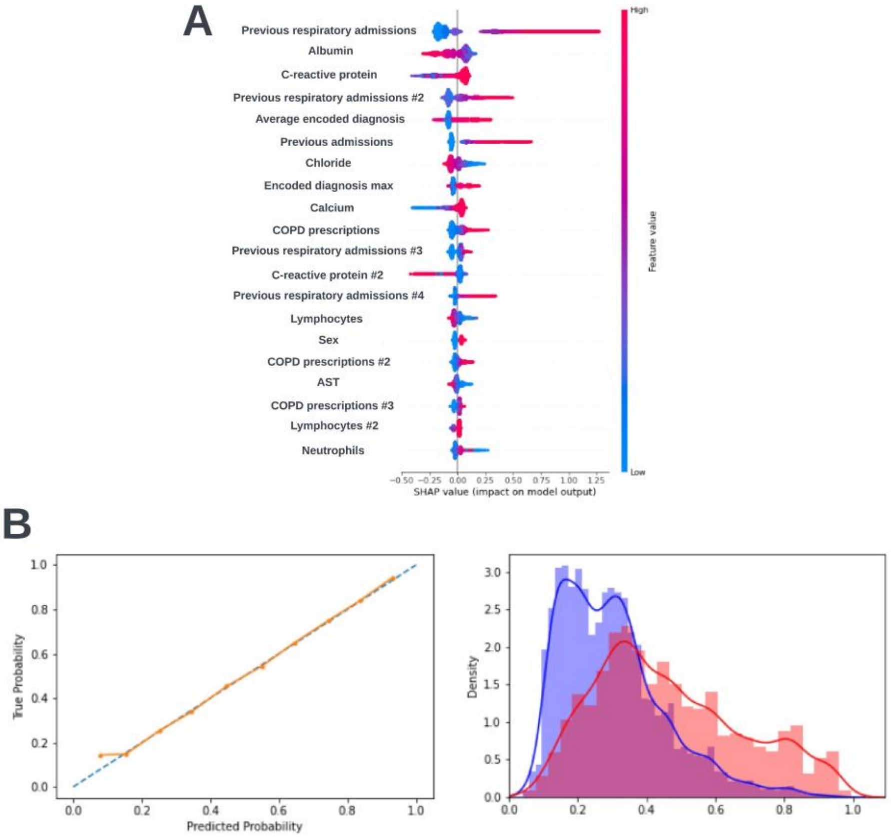

C

Group	Class	Precision	Recall	Positive selection	Validation % counts	Training % counts
SIMD 1	Readmission	0.68	0.40	21.5%	36.0	35.9
SIMD 2	Readmission	0.65	0.39	21.2%	18.9	18.9
SIMD 3	Readmission	0.71	0.38	18.9%	10.4	10.7
SIMD 4	Readmission	0.66	0.42	24.9%	8.4	8.1
SIMD 5	Readmission	0.68	0.46	25.6%	6.2	6.3
SIMD 6	Readmission	0.68	0.39	20.2%	4.7	4.7
SIMD 7	Readmission	0.62	0.30	15.8%	4.6	4.5
SIMD 8	Readmission	0.71	0.34	17.5%	3.7	4.0
SIMD 9	Readmission	0.67	0.21	9.6%	3.7	3.9
SIMD 10	Readmission	0.59	0.25	13.0%	3.3	3.1

Supplementary Figure 1. Screenshots showing different components of an illustrative model approval report. These reports are used to determine the acceptability of models for use within a live clinical environment within NHS Greater Glasgow & Clyde. A global SHAP plot with generalized feature names (A), a calibration plot and a probability distribution plot (B), and a table displaying performance metrics across SIMD subgroups (C) are shown.

Model approval checklist and notes

Model approval meeting notes

Model approval checklist

- Has the model gone through Lenus Engineering QA? ✓
- Are the training and holdout test cohorts independent and comparable? ✓
- Is the model formulation and algorithm suitable? ✓
- Are the model features: ✓
  - Appropriate for the intended use-case? ✓
  - Engineered appropriately? ✓
- Is the model calibration satisfactory? ✓
- Is the model performance satisfactory? In particular: ✓
  - The area under the precision recall curve (PR-AUC)? ✓
  - Expected numbers of patients brought forward correctly/incorrectly and missed? ✓
- Is the global model explainability bio-plausible? ✓
- Is the model fairness on different population sub-groups satisfactory? ✓

Summary
Model version: v1.1
Training date: 11/09/2023
Model approval meeting date: 02/10/2023
Model approval meeting outcome (approve): Yes

Supplementary Figure 2. A screenshot taken from an illustrative model approval report showing the checklist of requirements for a model to be approved for use within a live clinical environment within NHS Greater Glasgow and Clyde. Each item on the checklist must be met for a model to be approved.
